# State-Dependent Effective Connectivity in Resting-State fMRI

**DOI:** 10.3389/fncir.2021.719364

**Published:** 2021-10-27

**Authors:** Hae-Jeong Park, Jinseok Eo, Chongwon Pae, Junho Son, Sung Min Park, Jiyoung Kang

**Affiliations:** ^1^Department of Nuclear Medicine, Yonsei University College of Medicine, Seoul, South Korea; ^2^Department of Psychiatry, Yonsei University College of Medicine, Seoul, South Korea; ^3^Brain Korea 21 Project, Graduate School of Medical Science, Yonsei University College of Medicine, Seoul, South Korea; ^4^Center for Systems and Translational Brain Science, Institute of Human Complexity and Systems Science, Yonsei University, Seoul, South Korea; ^5^Department of Cognitive Science, Yonsei University, Seoul, South Korea

**Keywords:** effective connectivity, dynamic connectivity, resting state fMRI, ADHD, dynamic causal modeling (DCM)

## Abstract

The human brain at rest exhibits intrinsic dynamics transitioning among the multiple metastable states of the inter-regional functional connectivity. Accordingly, the demand for exploring the state-specific functional connectivity increases for a deeper understanding of mental diseases. Functional connectivity, however, lacks information about the directed causal influences among the brain regions, called effective connectivity. This study presents the dynamic causal modeling (DCM) framework to explore the state-dependent effective connectivity using spectral DCM for the resting-state functional MRI (rsfMRI). We established the sequence of brain states using the hidden Markov model with the multivariate autoregressive coefficients of rsfMRI, summarizing the functional connectivity. We decomposed the state-dependent effective connectivity using a parametric empirical Bayes scheme that models the effective connectivity of consecutive windows with the time course of the discrete states as regressors. We showed the plausibility of the state-dependent effective connectivity analysis in a simulation setting. To test the clinical applicability, we applied the proposed method to characterize the state- and subtype-dependent effective connectivity of the default mode network in children with combined-type attention deficit hyperactivity disorder (ADHD-C) compared with age-matched, typically developed children (TDC). All 88 children were subtyped according to the occupation times (i.e., dwell times) of the three dominant functional connectivity states, independently of clinical diagnosis. The state-dependent effective connectivity differences between ADHD-C and TDC according to the subtypes and those between the subtypes of ADHD-C were expressed mainly in self-inhibition, magnifying the importance of excitation inhibition balance in the subtyping. These findings provide a clear motivation for decomposing the state-dependent dynamic effective connectivity and state-dependent analysis of the directed coupling in exploring mental diseases.

## Introduction

At rest, the human brain exhibits temporal changes not only in the regional brain activity but also in the inter-regional coherence among distributed brain regions, called dynamic functional connectivity. A growing number of studies have explored dynamic functional connectivity (Chang and Glover, 2010; Cribben et al., 2012; Handwerker et al., 2012; Hutchison et al., 2013; Allen et al., 2014; Calhoun et al., 2014; Monti et al., 2014; Jeong et al., 2016; Jang et al., 2017; Park et al., 2018). Dynamic functional connectivity, however, does not provide information about the directed neural couplings among the brain regions, called effective connectivity (for review, see Park and Friston, 2013). Recently, Park et al. (2018) introduced the dynamic effective connectivity analysis to decompose the time courses of effective connectivity across consecutive windows into a set of dynamic effective connectivity components. To estimate the effective connectivity at each window of the resting-state functional MRI (rsfMRI) signals, they used spectral dynamic causal modeling (spDCM), which fits the cross-spectral density (CSD) (Friston et al., 2014) of the signals within the successive windows. The decomposition of the dynamic effective connectivity was achieved using the parametric empirical Bayes approach (PEB) (Friston et al., 2016) that models the effective connectivity of each window by incorporating random effects with a combination of multiple basis functions. This approach is an extension of a longitudinal study on recovery after thalamotomy in patients with essential tremors (Park et al., 2017). Van De Steen et al. (2019) applied the dynamic effective connectivity approach to the electroencephalogram (EEG) domain.

The dynamic effective connectivity analysis (Park et al., 2018) assumes continuous endogenous changes in the brain state. However, according to the nonlinear network theory, the brain transits spontaneously across multiple metastable states (Freyer et al., 2011, 2012; Rabinovich and Varona, 2011; Deco and Jirsa, 2012; Kelso, 2012; Cabral et al., 2014; Tognoli and Kelso, 2014; Deco et al., 2015; Breakspear, 2017). In dynamic functional connectivity analysis, the spontaneous transition of multiple states has been explored by clustering brain states according to functional connectivity patterns and evaluating the transition matrix (Allen et al., 2014; Damaraju et al., 2014). The Hidden Markov Model (HMM) with the multivariate autoregressive model (MAR) parameter for brain signals (HMM-MAR) is another type of connectivity-based approach to characterize organized state-transitioning in the brain network (Vidaurre et al., 2016, 2017). Here, the pattern of functional connectivity itself is considered a state, and the brain transits among multiple connectivity states. This dynamic connectivity approach is in contrast with nonlinear network models where the state transitions are modeled in terms of nonlinear coupling on fixed connectivity (e.g., Kang et al., 2019).

In this study, we extended the previous continuous dynamic effective connectivity method (Park et al., 2018) to state-dependent (not necessarily continuous) dynamic effective connectivity analysis. To decompose state-dependent effective connectivity, the hierarchical modeling of spDCM was conducted over the windows with the sequence of the brain states as regressors in PEB. This approach differs from Zarghami and Friston (2020), who proposed a full Bayesian scheme to estimate both transition property and state-dependent effective connectivity by placing priors on the transition probabilities among different connectivity states. Compared with Zarghami and Friston (2020), the current method is based on the hierarchical Bayesian linear modeling of each state and can be applied to decompose the state-dependent effective connectivity with diverse definitions of the brain states. For example, the brain states can be defined in terms of either regional activity patterns or the interaction patterns of rsfMRI, either at the target circuit or at any other circuit or the whole brain. Here, we identified the time courses of the brain states according to the MAR of the rsfMRI using the HMM. Since the MAR at a time window contains functional connectivity information at the window, the state transitions estimated by HMM-MAR can be viewed as relatively slow state dynamics of the inter-regional synchrony of fast regional dynamics.

The state-dependent analysis of effective connectivity is highly demanded in clinical brain research. Considering that the brain transits through different connectivity states even during a conventional period of rsfMRI acquisitions, the average connectivity across the entire time series may not fully represent the pathological state. This would be critical when the dysfunctionality of a diseased brain is sporadic. As a typical example, the ictal state of epilepsy is more representative of epilepsy than the inter-ictal state. Thus, we hypothesized that some temporal states may express abnormal mental diseases more than other brain states or a combination of all brain states. Furthermore, the dominant brain states may differ across individuals even in the same clinical diagnosis since the conventional diagnosis of psychiatric illnesses is generally based on subjective reports or behaviors (Insel and Cuthbert, 2015). If we consider the brain state in terms of functional connectivity, the connectivity at the dominant state and their occurrence characteristics can be a neurobiological biomarker to subtype mental diseases. Until now, several studies have suggested that connectivity states and their transitions are related to mental disorders such as schizophrenia (Damaraju et al., 2014), bipolar disorders (Rashid et al., 2014), autism (De Lacy et al., 2017), and attention deficit hyperactivity disorder (ADHD) (De Lacy and Calhoun, 2018). However, few studies have explored state-dependent and subtype-dependent connectivity characteristics in mental diseases.

In this study, we proposed a framework to estimate state-dependent effective connectivity. In this framework, we characterized slow transitions in context- or state-dependent effective connectivity among nodes, where the slow transitions generate changes in the fast neuronal dynamics under a standard dynamic causal model (DCM) of cross-spectral activity. We first assumed a forward model with slow transitions among a small number of intrinsic connectivity states. Each connectivity state then generates a fast neuronal activity under the dynamic causal model with the state-specific effective connectivity. To invert this forward model, the connectivity state at each time point was identified using an HMM-MAR of the observed time series. The effective connectivity was estimated using spDCM for overlapping time windows of fixed size. Because connectivity state transitions may occur within each window, we modeled the DCM estimates of effective connectivity with a mixture of connectivity states based upon their occupation times. These occupation times were used as regressors to explain the DCM of effective connectivity (i.e., parameters) in a (Bayesian) general linear model (GLM) within the PEB framework. Using PEB, we inferred the intrinsic effective connectivity that generates the time courses of the observed connectivity states. This hierarchical modeling was implemented on PEB with one (or more) GLM at the higher levels and a DCM at the lowest level. Note that the data are used twice in this modeling. First, the data were used to furnish proxies for different connectivity states and their time-dependent transitions at a slow timescale. The same data were used to estimate the effective connectivity, within short windows, at a fast timescale. The two timescales were integrated within a hierarchical (PEB) model of dynamic effective connectivity. We extended this hierarchical procedure to the group-level analysis by adding another step of PEB. One issue in the state-dependent/subtype-dependent effective connectivity studies of multiple subjects (or groups) is identifying the connectivity states that are conserved over individuals. In other words, one needs to identify a particular connectivity state that has the same meaning in every subject. We achieved this by performing a cluster analysis of (MAR-based) functional connectivity from all the individuals together. By doing so, the transitions among particular connectivity states could be meaningfully interpreted over individuals. And, crucially, the group differences in state-specific effective connectivity could be quantified.

To test its usefulness, we applied the proposed method to characterize ADHD, one of the complex neurobehavioral disorders commonly observed in childhood. We focused on the ADHD-combined type (ADHD-C), one of the most common subtypes in early ADHD, showing inattentiveness and hyperactivity/impulsivity. As the brain connectivity changes significantly with age, we focused only on the early years of ADHD-C (ages 7–10) and age-matched typically developed children (TDC). According to the brain connectivity analyses on ADHD (see Saad et al., 2020 for review), we concentrated on the default mode network (DMN) (Sonuga-Barke and Castellanos, 2007; Zang et al., 2007; Fair et al., 2010; Qiu et al., 2011; Brown et al., 2012; Anderson et al., 2014; Barber et al., 2015). This study aims to show the plausibility of decomposing state-dependent dynamic effective connectivity and the advantage of the state-dependent analysis of the effective connectivity of the brain in subtyping and characterizing mental diseases.

## Background

The procedures were composed of the following steps: (1) extraction of time series from the DMN and whole-brain; (2) identification of connectivity states and their transitions using HMM; (3) estimation of the first level spDCM for all the windows at each subject; (4) second-level state-dependent effective connectivity estimation using PEB with the states driven from the HMM; (5) group comparison using PEB. We extracted the time series of DMN using the group independent component analysis (gICA) of the rsfMRI.

More specifically, the analysis comprises the following steps: (1) the time series were clustered into state vectors using HMM; (2) the time series were segmented into multiple windows with a regular step, and the spDCM was estimated for each window. For HMM, we defined a brain state using MAR, equivalent to the cross-spectrum (Fourier transform of the cross-correlation) among the brain regions. The slow changes in the effective connectivity (estimated by spDCM at consecutive windows) were modeled with a GLM using the occupation proportions of the brain states as regressors in the second-level PEB analysis. Finally, the third level PEB was applied to study the group characteristics of ADHD-C.

### Hidden Markov Modeling (HMM) for State Estimation

We used the HMM-MAR toolbox (https://github.com/OHBA-analysis/HMM-MAR) (Vidaurre et al., 2016) and applied HMM to the time series with MAR of order 5, which was used in the spDCM estimation in this study. From the HMM of the observed vector time series ***y***(t) and *z*_*t*_ the hidden state variable, we derived the state probability time series *s*_*k*_(*t*) for the state indices *k* ∈ {1, 2, ⋯ , *K*} and time *t* = 1, 2, ⋯ , *T*.

The observation model is denoted as


(1) $$\begin{array}{r} y_{t}|z_{t}=k\sim N\left(\underset{l\in A}{\sum }{\text{y}}_{t-1}{\text{W}}_{l}^{\left(k\right)},\Sigma^{\left(k\right)}\right),\\ p\left(z_{t}=k|\text{Y}\right)\equiv s_{k}\left(t\;\right),\\ P\left(z_{t}=k_{1}|z_{t-1}=k_{2}\right)\equiv \Theta_{k_{1}k_{2}},P\left(z_{1}=k\right)\equiv \;\eta_{k}. \end{array}$$

The HMM-MAR estimates the model parameters of Θ_*k*_1_*k*_2__ and η_*k*_, and uses these results to estimate *s*_*k*_ (*t*). See Vidaurre et al. (2016) for details.

For each subject, we segmented the whole time series ***y***(t) into multiple consecutive windows. We defined a state occupation index $u_{k}^{\left(i\right)}$ for a state *k* at the *i*-th window as the average of the state probability *s*_*k*_ at the window,


(2) $$\begin{array}{l} u_{k}^{\left(i\right)}=\underset{t\in w_{i}}{\sum }s_{k}\left(t\right)/\underset{t\in w_{i}}{\sum }1. \end{array}$$

This occupation index was used to denote brain states in the subsequent analysis. Since MAR reflects the functional connectivity of the window, we called the state defined by the HMM-MAR as the “connectivity state” or “functional connectivity state” in this paper.

### State-Dependent Spectral DCM

The spDCM models the cross-spectra of the resting-state endogenous fluctuations (in the absence of external input) using a neuronal dynamics model with the effective connectivity matrix *A* as a parameter set and a hemodynamic response model *h*.


(3) $$\begin{array}{l} ẋ\left(t\right)=Ax\left(t\right)+v\left(t\right), \end{array}$$

(4) $$\begin{array}{l} y\left(t\right)=h\left(x\left(t\right),\theta_{h}\right)+e\left(t\right),\;e\;\sim \;N\left(0,\;\Sigma \right) \end{array}$$

where *x* and *y* represent a vector of hidden neural states and the observed fMRI signals at the brain regions. The endogenous neural fluctuations are indicated by *v*(t*)* while *e*(t) represents an observation error. The fMRI signal *y* is approximated with a nonlinear hemodynamic response function *h* of hidden neuronal states *x*(t) with parameters θ_*h*_ (Stephan et al., 2007). In spDCM, the intrinsic effective connectivity (i.e., the *A* matrix) was estimated using a Bayesian model inversion in the spectral domain after transforming the observed signal ***y*** into the cross-spectra. The details can be found in Friston et al. (2014) and Razi and Friston (2016). To characterize state-dependent effective connectivity, we estimated the spDCMs for the equally partitioned *W* windows.


(5) $$\begin{array}{l} {ẋ}_{\left(i\right)}=A_{i}x_{\left(i\right)}+v_{i}\\ \;=\left(A_{0}+\overset{K}{\underset{k=1}{\sum }}A_{\left(k\right)}u_{k}^{\left(i\right)}\right)x_{\left(i\right)}+v_{i},\;for\;i=1,\cdot \cdot \cdot ,W\\ \;=\;\left(\left[1\;u_{1}^{\left(i\right)}\;u_{2}^{\left(i\right)}{\cdot \cdot \cdot \;u}_{K}^{\left(i\right)}\right]{\left[A_{0}\;A_{\left(1\right)}\;A_{\left(2\right)\;}\cdot \cdot \cdot A_{\left(K\right)}\right]}^{t}\right)x_{\left(i\right)}+v_{i}\\ \;=\left(X_{1}\beta \right)x_{\left(i\right)}+v_{i} \end{array}$$

The effective connectivity for the *i*-th window is A_*i*_, which can be decomposed into state-dependent components. The effective connectivity for the *i*-th window can be modeled with a combination of *K* state-dependent contribution $u_{k}^{\left(i\right)}$ (a column of the design matrix *X*_1_) and their corresponding state-dependent effective connectivity matrices A_(*k*)_ (a state-dependent matrix in the subject's parameter set β). Under this model, we used PEB to find A_(*k*)_ for the given state sequence $u_{k}^{\left(i\;\right)}$.

### PEB Estimation of Dynamic Effective Connectivity

Technically, the current approach extends the dynamic effective connectivity analysis that explored the continuous endogenous fluctuations of the effective connectivity components (Park et al., 2017). The dynamic effective connectivity analysis was based on PEB (Friston et al., 2015, 2016), with a three-level hierarchical model: (1) window level, (2) subject level, and (3) group level. Specifically, at the first window level, we inverted the spDCMs for all the windows of each subject. We used a fully connected intrinsic connectivity model of spDCMs. The state-dependent effective connectivity was modeled at the second level with state-dependent regressors *X*_1_ across the windows for each subject. This hierarchical PEB model can be described with the following equations:


(6) $$\begin{array}{l} y_{ij}=\Gamma \left(A_{ij}\right)+\varepsilon_{ij}^{\left(1\right)},\;\varepsilon_{ij}^{\left(1\right)}\;\sim \;N\left(0,\Sigma^{\left(1\right)}\right) \end{array}$$

(7) $$\begin{array}{l} A_{ij}=X_{1}\beta_{j}^{\left(2\right)}+\varepsilon_{j}^{\left(2\right)},\;\varepsilon_{j}^{\left(2\right)}\;\sim \;N\left(0,\Sigma^{\left(2\right)}\right) \end{array}$$

Equation (6) states a generative model of the observed fMRI signal *y*_*ij*_ at the *i*-th window of the *j*-th subject with a function Γ of the effective connectivity *A*_*ij*_ (at the *i*-th window of the *j*-th subject) plus independent and identically distributed (i.i.d.) observation noise $\varepsilon_{ij}^{\left(1\right)}$. The parameters of this model *A*_*ij*_ correspond to *A*_*i*_ in Eq. (5) for the *j*-th subject. The parameters of the *j*-th subject (effective connectivity) is $\beta_{j}^{\left(2\right)}$. *X*_1_ is a design matrix that express the brain states of all the windows (the column of *X*_1_ is composed of the occupation index of each state derived from HMM-MAR). *A*_*ij*_ is expressed with the parameters that were obtained as the parameters of the subject with a designed matrix *X*_1_ and random effects $\varepsilon_{j}^{\left(2\right)}$. In the spDCM, Eq. 6 was solved in the cross-spectral domain by transforming the observed *y*_*ij*_ into CSD. For details, see Friston et al. (2014) and Razi and Friston (2016).

**Figure 1:**
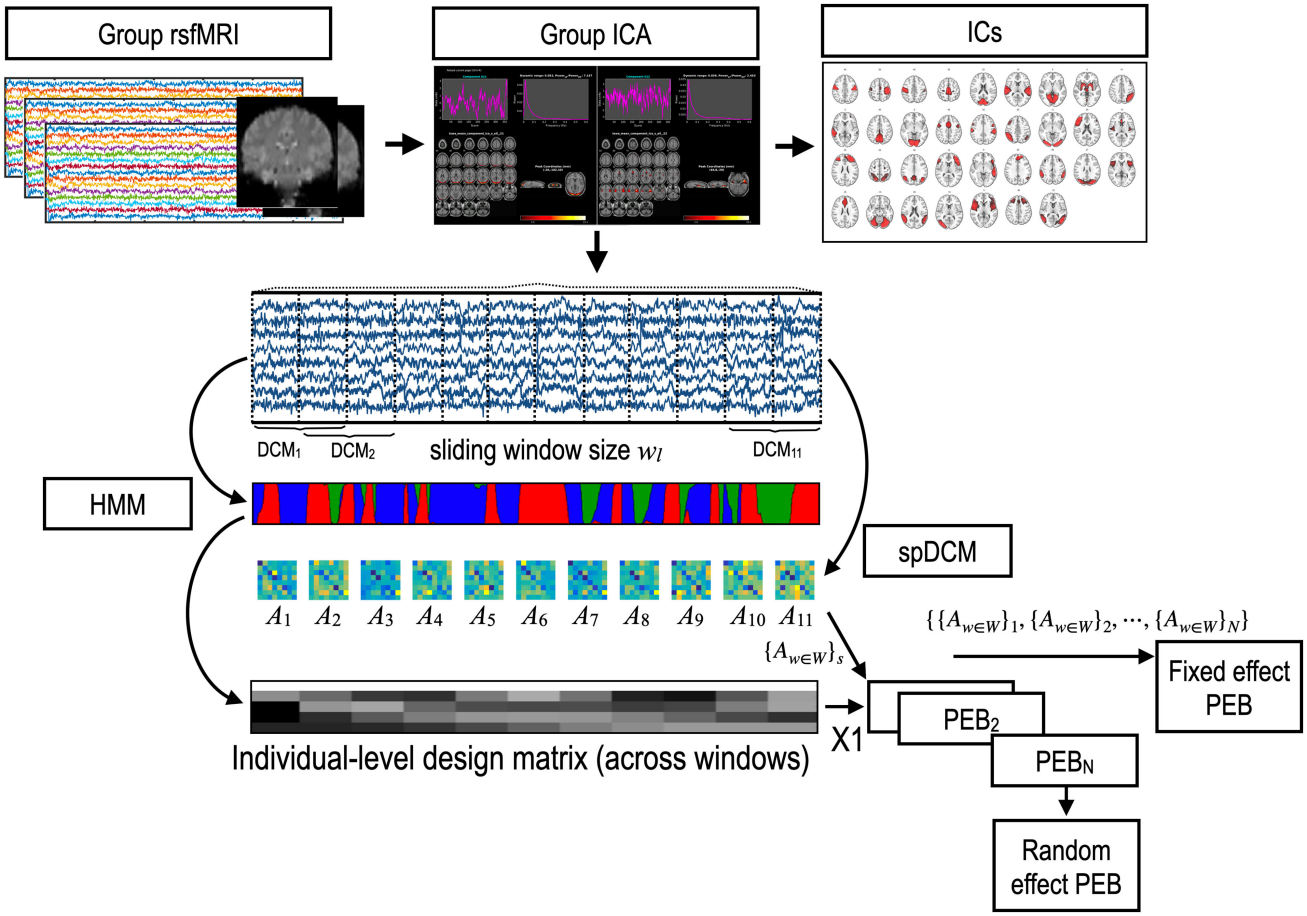
Procedures for state-dependent effective connectivity analysis. A time series of the brain activity was obtained using a group independent component analysis (gICA) of the resting-state functional MRI (rsfMRI). A spectral DCM (spDCM) was estimated for each window separately for each participant. Parametric empirical Bayesian analysis (PEB) with temporal regressors of states can estimate the state-dependent effective connectivity in each individual. For the group-level analysis, the fixed effects model concatenates sets of windowed DCM parameters ({_*A*_*w*∈*W*_}*s*_) of all individuals into a set of DCM parameter series, which were then modeled with state labels using PEB. The random-effects model can be solved by applying PEB two times: one time for the windowed DCMs of each individual; and other time for the parameters of each individual, estimated using the first-level PEB to infer the group-level parameter sets. W:{1,2, …, number of windows}, N: number of subjects.

Group-level modeling can be achieved either by the fixed-effects model or the random-effects model (see Figure 1).

In the random-effects model, the state-dependent effective connectivity of each subject $\beta_{j}^{\left(2\right)}$were modeled at the group level using a (Bayesian) GLM with group-common and/or group-difference parameters β^(3)^ and their regressors *X*_2_ according to the diagnosis of the subject.


(8) $$\begin{array}{l} \beta_{j}^{\left(2\right)}=X_{2}\beta^{\left(3\right)}+\varepsilon^{\left(3\right)},\;\varepsilon^{\left(3\right)}\;\sim \;N\left(0,\Sigma^{\left(3\right)}\right) \end{array}$$

(9) $$\begin{array}{l} \beta_{j}^{\left(3\right)}=\varepsilon^{\left(4\right)},\;\varepsilon^{\left(4\right)}\;\sim \;N\left(0,\Sigma^{\left(4\right)}\right) \end{array}$$

Equation (8) states that each state-dependent connectivity $\beta_{j}^{\left(2\right)}$of the subject were generated from a group level model with a design matrix *X*_2_ and group parameters β^(3)^. The group parameters β^(3)^ were sampled randomly with a zero-mean Gaussian and covariance matrix Σ^(4)^. Within this hierarchy, the first-level models were spDCMs, whereas the second- and third-level models were Bayesian GLMs.

We implemented state-dependent effective connectivity according to this hierarchical model using two levels of PEB (Figure 1): (1) PEB of DCMs across windows for each subject with a design matrix *X*_1_ and (2) PEB of individual effect sizes (i.e., state-dependent connectivity) across subjects, with a design matrix *X*_2_ for group inference (i.e., PEB of PEB).

In the fixed-effects model, the implementation was much simpler than the random-effects model by concatenating $\theta_{ij}^{\left(1\right)}$ across the windows and subjects by assuming no differences in the state-dependent effective connectivity across the subjects. For the group comparison, we estimated the PEB for each group based on the fixed-effects model, which was then evaluated by an additional PEB with group contrasts for the group-level inference.

## Materials and Methods

To establish the face validity of the state-dependent effective connectivity analysis, we first performed a simulation study to see if we could recover known (simulated) changes in the dynamics of effective connectivity. To establish predictive validity, we then applied the analysis to empirical data using the ADHD-C and TDC datasets.

### Simulation for State-Dependent DCM

**Figure 2:**
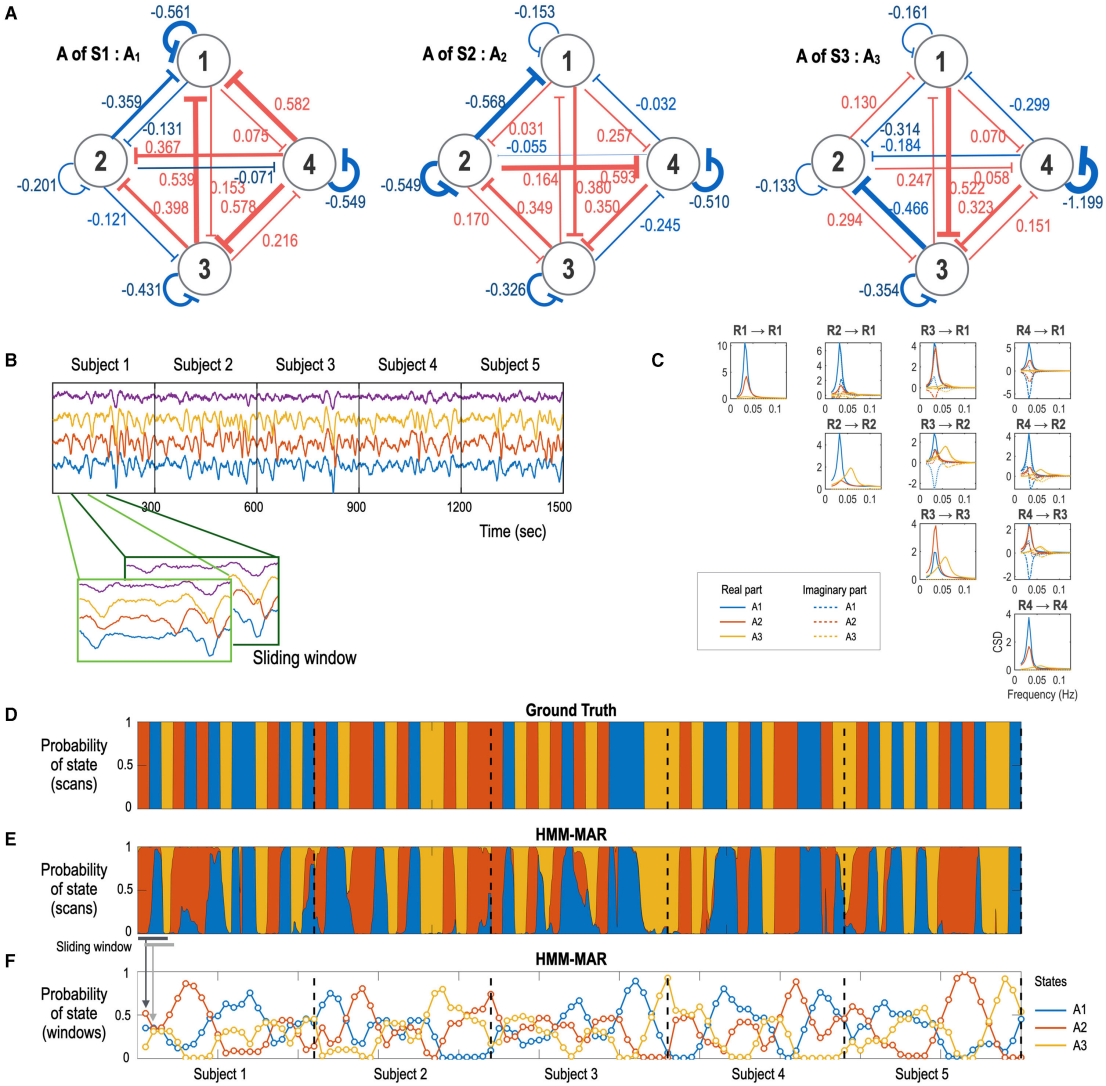
Simulation study with a time series generation and state-sequence estimation using HMM-MAR. **(A)** A network system with three different effective connectivity states, A1 (A matrix in Eq. 3 for the state S1), A2 (A matrix for the state S2), and A3 (A matrix for the state S3), used in this simulation are displayed. **(B)** fMRI signals with 300 s length (TR = 0.8 s, total 375 samples) were generated according to the dynamic equation in Eqs. 3–4 for five subjects by transitioning the three effective connectivity matrices. **(C)** the cross-spectral density for the three effective connectivity states is presented in blue, red, and yellow colors for A1, A2, and A3, respectively. **(D, E)** The ground-truth state sequence **(D)** and the estimated state sequence **(E)** obtained by the HMM-MAR analysis with *K* = 5 and order = 5. Only three clusters dominated the entire time for all the subjects. The blue, red, and yellow represent the three dominant effective connectivity states A1, A2, and A3. The occupation indices (or occupation times divided by the window size) of the three major states for each sliding window for **(E)** are displayed at **(F)**. The state-dependent effective connectivity analysis is based on these estimated occupation indices as a state sequence.

In the simulation setting, three brain connectivity states of a network with four nodes were considered. To create a biologically plausible model, we used the effective connectivity matrices estimated from the DMN time series of randomly selected three healthy subjects using spDCM. The effective connectivity matrices, *A1*, and *A2*, and *A3* were (see Figure 2A)


(10) $$\begin{array}{l} A1=\left[\begin{array}{cccc} -0.561 & -0.359 & 0.539 & 0.582\\ -0.131 & -0.201 & 0.398 & 0.367\\ 0.153 & -0.121 & -0.431 & 0.578\\ 0.075 & -0.071 & 0.216 & -0.549 \end{array}\right],\; \end{array}$$

(11) $$\begin{array}{l} A2=\left[\begin{array}{cccc} -0.153 & -0.568 & 0.164 & -0.032\\ 0.031 & -0.549 & 0.349 & -0.055\\ 0.380 & 0.170 & -0.326 & 0.350\\ 0.257 & 0.593 & -0.245 & -0.510 \end{array}\right],\; \end{array}$$

(12) $$\begin{array}{l} A3=\left[\begin{array}{cccc} -0.161 & 0.130 & 0.247 & -0.299\\ -0.314 & -0.133 & -0.466 & -0.184\\ 0.522 & 0.294 & -0.354 & 0.323\\ 0.070 & 0.058 & 0.151 & -1.199 \end{array}\right].\; \end{array}$$

Using the forward model described in Eqs. 3 and 4, we generated 375 sampled fMRI signals (TR = 0.8 s, 5 min length) for five subjects using the integrator spm_int_J.m and hemodynamic response function spm_gx_fmri.m in the Statistical Parametric Mapping (SPM) toolbox (https://www.fil.ion.ucl.ac.uk/spm/) (Figure 2B). Each state persists for 20 s, followed by a state transition according to a transition matrix, *TM*,


(13) $$\begin{array}{l} TM=\;\left(\begin{array}{ccc} 0.3 & 0.5 & 0.1\\ 0.2 & 0.2 & 0.8\\ 0.5 & 0.3 & 0.1 \end{array}\right). \end{array}$$

Here, the element *TM*_*ij*_ represents the transition probability from state *j* to state *i*. The cross-spectra for the three states is presented in Figure 2C.

For the time series generated by a state transitioning network (Figure 2D), we performed an HMM-MAR analysis with the maximal numbers of states, *K* = 5, and MAR order of 5 with 300 different initial values using the HMM-MAR toolbox (https://github.com/OHBA-analysis/HMM-MAR) (Vidaurre et al., 2016). Although we set *K* = 5, most state sequences have two or three states at most. We selected a state sequence that contains the three alternating states and used the sequence to compose a state regressor in the state-dependent effective connectivity analysis using PEB (Figures 2E,F).

Based on the time series and sequence of states, we estimated the state-dependent effective connectivity. We divided the entire time series (375 samples) into multiple windows with a window size of 75 samples (60 s) and an overlap of 45 samples (36 s). For each window, we conducted an spDCM, which resulted in 22 spDCM series for a subject. Using a fixed-effects model, we performed a group-level PEB analysis for all the spDCMs obtained from 110 windows (22 windows × 5 subjects). The state occupation indices for the dominant states of each window were used as a regressor in the PEB analysis.

**Figure 3:**
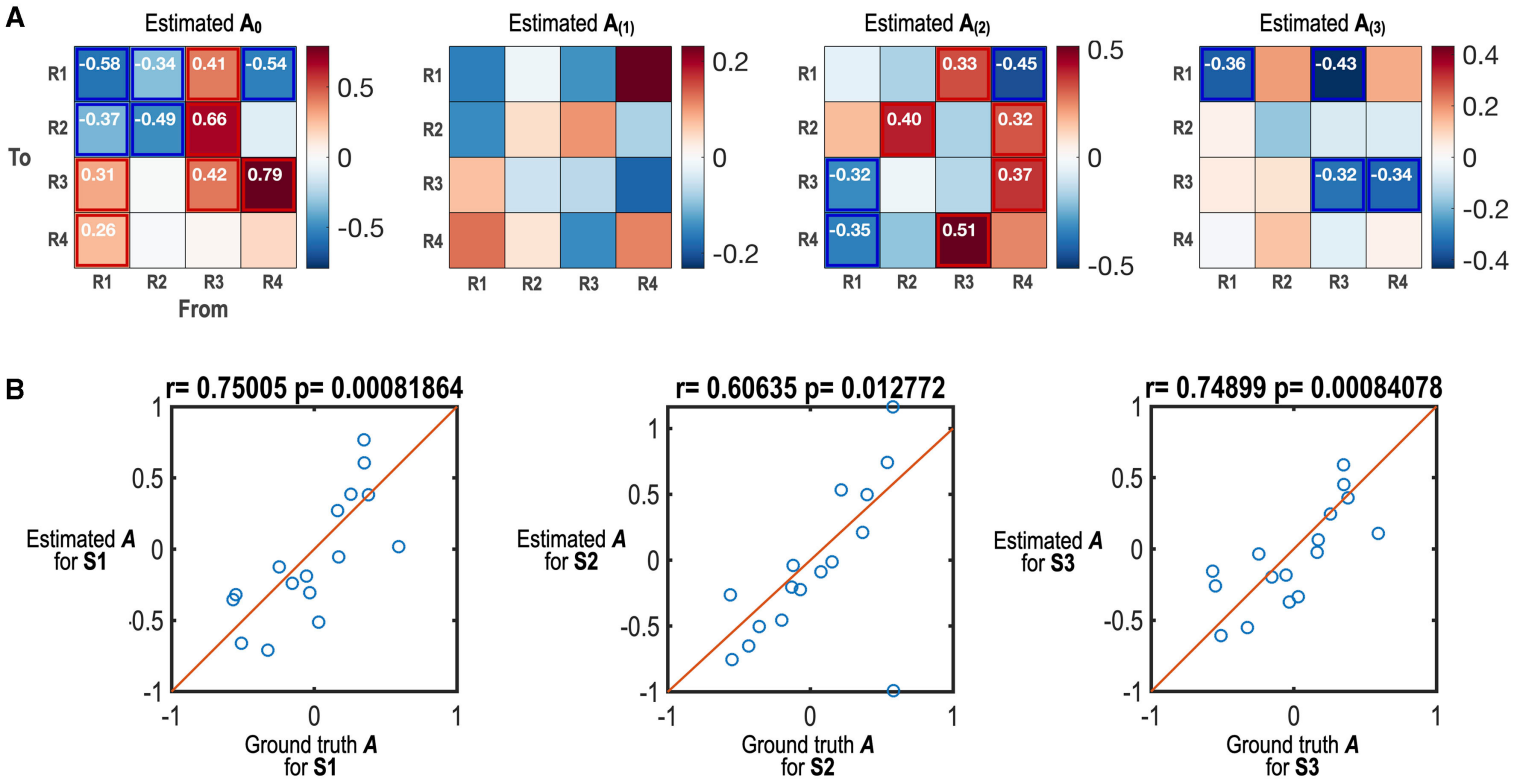
Simulation results of the state-dependent effective connectivity estimation using HMM-MAR and spDCM. **(A)** The estimated state-dependent effective connectivity matrices (maximum a posteriori probability estimate; MAP) are displayed. **A**_**0**_ indicates the offset or baseline effective connectivity for states S1, S2, and S3. **A**_**(1)**_, **A**_**(2)**_ and **A**_**(3)**_ indicate the MAPs corresponding to the regressors for S1, S2, and S3. The effect sizes that survived the criterion of 95% posterior confidence are shown in numbers and rectangles. **(B)** The scatter plots between estimated state-dependent effective connectivity and those of the ground truth are displayed. Estimated A for S1, S2, and S3 are a sum of **A**_**0**_ and **A**_**(1)**_, a sum of **A**_**0**_ and **A**_**(2)**_ and a sum of **A**_**0**_ and **A**_**(3)**_, respectively. Among the 48 network parameters, 38 parameters (~80%) of the true parameters were in the range of 95% credible intervals of estimated parameters.

Figure 3 shows the results of the estimated effective connectivity for the three primary states. The extracted effective connectivity parameters were highly correlated with the true connectivity parameters (*r* = 0.6–0.75, *p* < 0.01) (Figures 3A,B). Among the 48 connectivity parameters, 38 effective connectivity of the ground truth (~80%) were in the range of 95% credible intervals of the estimated effective connectivity. This result denotes the reliability of the proposed method in estimating the state-dependent effective connectivity. We also conducted the same analysis using the random-effects model, which showed slightly less accuracy than the fixed-effects model.

### Experiment for State-Dependent DCM Using ADHD Dataset

#### Dataset and Image Processing

To decompose the state-dependent effective connectivity in the DMN of ADHD, we analyzed the rsfMRI data from the Healthy Brain Network (HBN) database (Alexander et al., 2017). The dataset was obtained from the HBN Biobank of the Child Mind Institute, releases 1–6 (http://fcon_1000.projects.nitrc.org/). From the subjects that completed both the MR sessions and clinical assessment, we selected children with ADHD-C and TDC. Since ADHD-C and TDC are under developmental state, to reduce the age effect, we narrowed the age range between 7 and 10. This age range corresponds to grades 2–5 of schooling. We screened the rsfMRI and T1-weighted structural MRI and excluded subjects with excessive motion or poor data quality. The resulting dataset includes 56 children with ADHD-C (mean age of 8.8 and standard deviation of 1.06 years, 42 males and 14 females), and 32 children with TDC (mean age of 8.7, a standard deviation of 1.06, 15 males and 17 females).

For the ADHD-related behavioral scores, we used the subscales of the Child Behavior Checklist (CBCL) (Achenbach and Rescorla, 2001), more specifically, the CBCL score of internalizing problems (CBCL-INT) and the CBCL score of externalizing problems (CBCL-EXT). The CBCL-INT is a sum of the anxious/depressed, withdrawn-depressed, and somatic complaints sub-scales in CBCL, while CBCL-EXT is the sum of the rule-breaking and aggressive behavior sub-scales. We also used the attention problems score (CBCL-AP).

All the symptom-related scores of CBCL-EXT, CBCL-INT, and CBCL-AP in this study show significant statistical differences between ADHD-C and TDC (*p* < 0.0000). Besides the attention-related scores, there was a significant group difference in the percentile of the full-scale intelligence quotient (FSIQ) of the Wechsler Intelligence Scale for Children (WISC) (Mean: 43.9 and std: 33.6 for ADHD-C; Mean: 61 and std: 31.1 for TDC; *p* = 0.02). Therefore, we used FSIQ, sex, and age as nuisance variables to regress out in the correlation analysis and group comparison.

We followed a typical rsfMRI preprocessing steps using SPM12 (http://www.fil.ion.ucl.ac.uk/spm) (Friston et al., 1995) and an inhouse MATLAB (Mathworks, Inc., Natick, Massachusetts, United States) toolbox, MNeT (multimodal network analysis tool, http://neuroimage.yonsei.ac.kr/mnet/). We discarded the first five volumes of the resting-state scans and excluded the scans with more than 365 volumes to match the number of volumes across all subjects. The processing step includes slice time correction, motion correction, co-registration to T1-weighted images, and spatial normalization to register the images to the Montreal Neurological Institute (MNI) template using nonlinear transformation.

**Figure 4:**
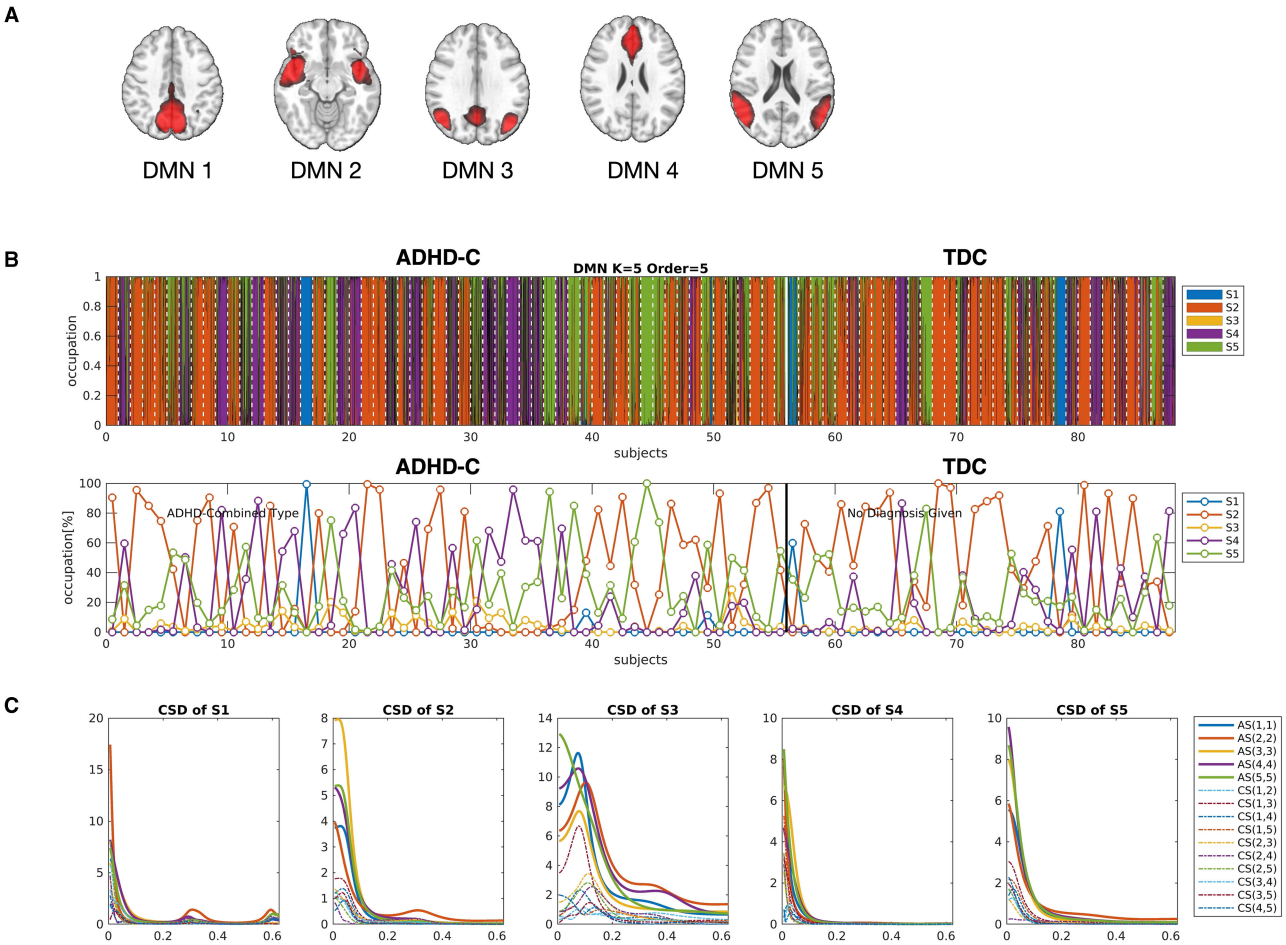
**(A)** The five independent components were used as nodes for the DMN network in the current study. **(B)** The occupation indices of five major cross-spectral density (CSD) states for all individuals, derived from HMM-MAR (*K* = 5 and order = 5), were concatenated and colored according to the amount of occupation portion of each state at each window. The states for individuals (21 windows per individual) were sectioned by white dotted lines. The lower panel of **(B)** indicates the occupation indices of all the states in each individual. **(C)** The auto-spectra (AS) and cross-spectra (CS) densities for the five major states were displayed. Among the five major states, S2, S4, and S5 were shared by all subjects.

After preprocessing the data, we performed gICA using the Group ICA of fMRI Toolbox (GIFT) (https://trendscenter.org/software/gift/) (Calhoun et al., 2001). The number of independent components was estimated as 41 according to the minimum description length (MDL) criterion (Li et al., 2007). After reducing all the rsfMRI data into 41 dimensions using the principal component analysis (PCA), we conducted the Infomax ICA algorithm (Himberg et al., 2004) and selected the best run from 100 runs to identify the group independent components. The subject-specific spatial maps and corresponding time courses were estimated using back-reconstruction. The time courses of independent components were converted to z-scores. After visual inspection, five components were classified as the DMN (Figure 4A). We also chose noise-free 38 whole-brain independent components. Since an HMM with a high MAR order is not suggested for the large network size for it has too many model parameters to estimate, we first conducted a dimension reduction of all the whole-brain components in both groups using PCA. We chose the number of components to be 11 to compromise the explainability and the size of the time series for the HMM. The 11 components explained 70% of the data used in the current study. We mainly focused on the state-dependent effective connectivity at the DMN using a state sequence derived from the HMM-MAR of the DMN time series. The whole-brain time series was used as a state sequence for the state-dependent effective connectivity analysis of the DMN as an alternative example of the current state-dependent effective connectivity scheme.

In summary, we constructed two multivariate time series. The first, based upon the default mode comprised five independent components. The second, based upon whole-brain responses, comprised 11 principal components. We apply the following analysis to both time series, focusing on the DMN time series.

We identified the brain states using HMM-MAR for the DMN time series. We used the order of MAR as 5 for both HMM and DCM. The number of maximal states was set to *K* = 5 according to the previous dynamic functional connectivity study of the DMN (De Lacy and Calhoun, 2018). We then analyzed the occurrences of the five states across individuals. Among the five states, we chose only three states that were shared by all the subjects (Figure 4). Thus, the subsequent analysis was based on these three dominant states.

We partitioned the whole time series into multiple consecutive windows with the window size and overlap size. Since the model and data themselves differ with the number of windows, a direct comparison of the model evidence is inappropriate in the PEB framework. Thus, we followed the approach Zarghami and Friston (2020) proposed, which determines the optimal window size by maximizing the relative log evidence of a dynamic state model compared with a stationary model for a given window size. Based on the three dominant states, we optimized the window size and overlap size by evaluating the log Bayes factor (BF) for 12 pairs of window size and overlap size compared with its stationary counterpart (only a single state throughout the time series). The 12 pairs of window and overlap sizes were (window size = 45, overlap size = 30), (45, 15), (60, 45), (60, 30), (75, 60), (75, 45), (90, 75), (90, 60), (120, 105), (120, 90), (150, 135), and (150, 120). Overlap sizes were chosen to make sliding window steps (window size – overlap size) 15 or 30. For example, for window size 60 and overlap size 45, the sliding step is 60 – 45 = 15. The spDCMs were estimated for the Hanning-windowed samples for all the windows with different window sizes and overlapping sizes. The state occupation indices were also evaluated over those windows, which were used as regressors in the PEB. As a fixed approach, we concatenated the spDCMs for all the windows and subjects and conducted PEB with the state occupation indices of the three major states as regressors. We also conducted PEB without any state regressors (equivalent to averaging spDCMs as a stationary model). For each pair of window size and overlap size, the free-energy of PEB with state regressors was compared with that without any state regressors in the log space. The window size and overlap size that had the maximal log BF was chosen for the subsequent analysis.

We then subtyped the individuals according to their state occupation patterns using a modularity optimization scheme. We evaluated the ADHD-related score differences in terms of the subtypes and diagnostic groups. For all the windows of each subject, we estimated the spDCMs, followed by the state-dependent decomposition of the effective connectivity. We compared the state-dependent effective connectivity across diagnostic groups (ADHD-C and TDC) according to the subtypes at each state of functional connectivity. We also compared the effective connectivity difference in the children with ADHD-C in different subtype groups. To show the applicability of the current method to different definitions of states, we conducted the same analysis with states defined with the interactions among the whole brain independent components.

## Results

**Figure 5:**
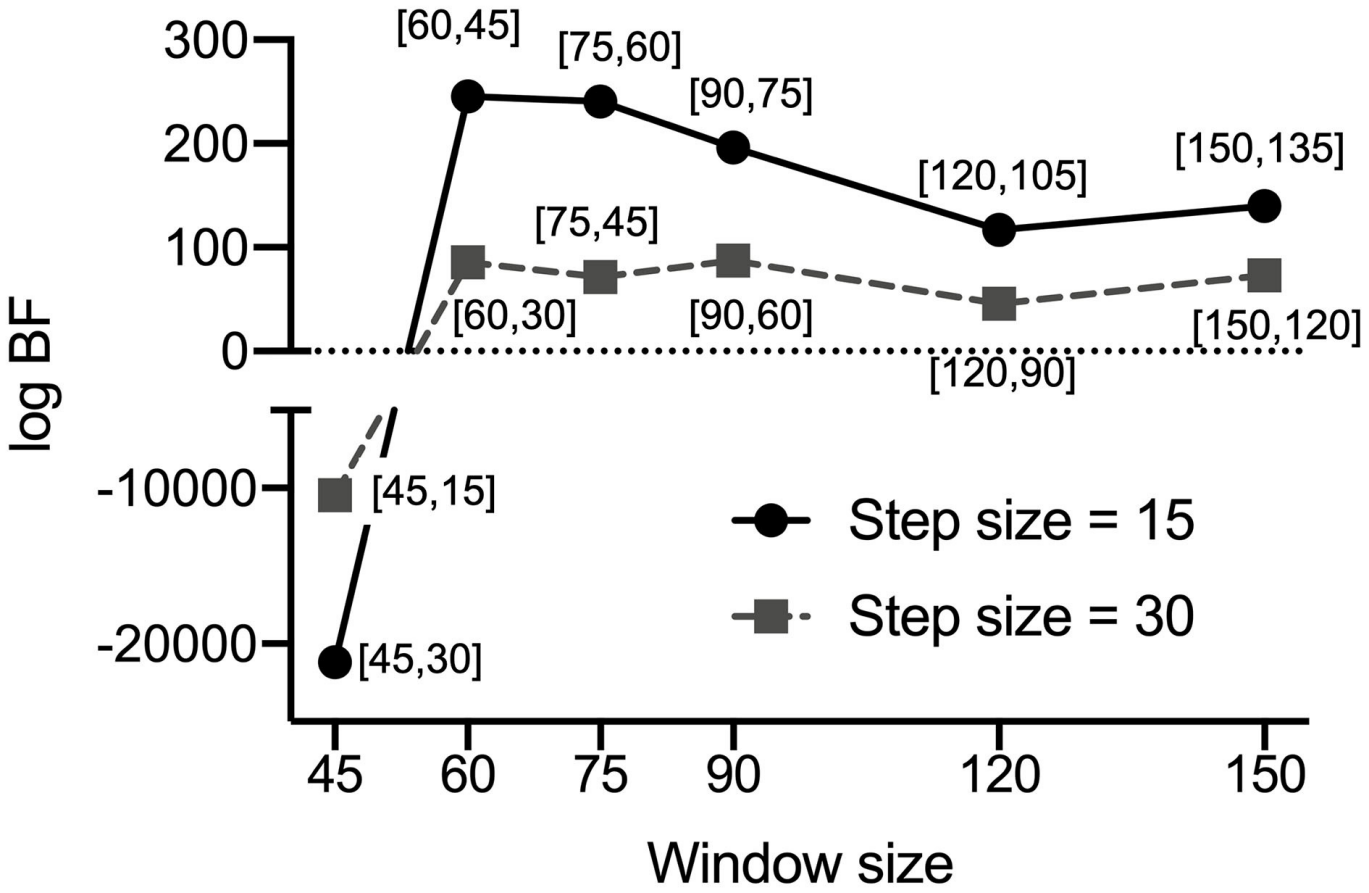
Window size and overlap size-dependent log Bayes factors (BF) for the experimental data. The log BFs of the 12 pairs of window sizes and overlap sizes in the PEB analysis are presented. According to Zarghami and Friston (2020), we determined the optimal window size and overlap size by maximizing the relative log evidence (or the log BF) of a dynamic state model compared with a stationary model for a given window size and overlap size in the PEB analysis. The bracket contains a pair [window size, overlap size]. Among the 12 pairs of window size and overlap size, the window size = 60 and overlap size = 45 showed the highest BF in the DMN of the current experimental data. Overlap sizes were chosen to make sliding window steps (window size minus overlap size) 15 or 30 (see Method section in detail).

Figure 4 displays the results of state distribution when we decomposed the entire time series of the DMN of all the subjects using HMM-MAR. As shown in Figure 4, most states belong to states S2, S4, and S5. Thus, we used the states S2, S4, and S5 in the subsequent analysis. Figure 5 shows the window-size and overlap-size dependent log BFs, on which we based the window size = 60 (48 s) and overlap size = 45 (36 s) in the current experimental study. The model parameter estimation accuracy according to window size is presented in Supplementary Appendix 1.

**Figure 6:**
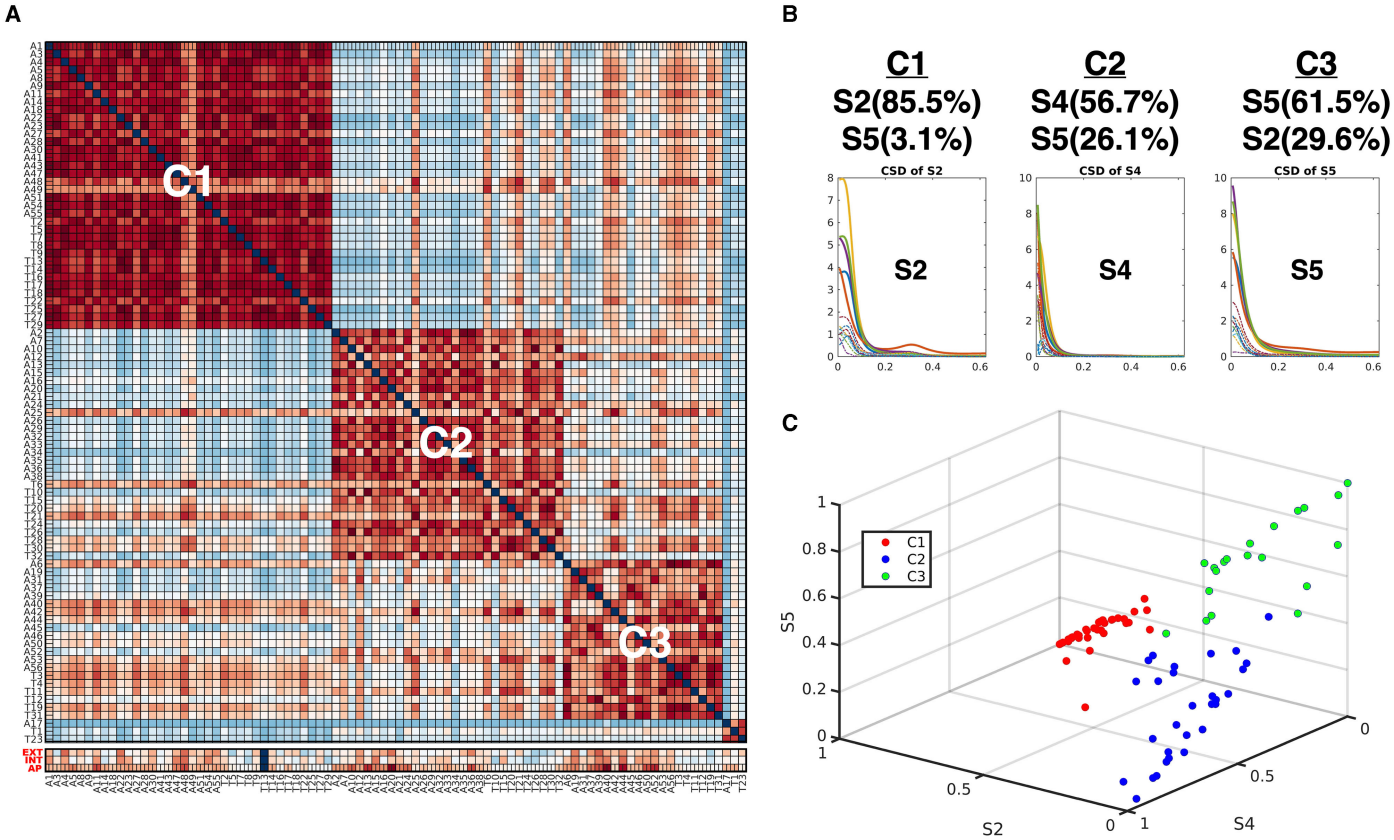
**(A)** Subtypes of individuals according to the similarity of the occupation indices of an individual of the five connectivity states. The similarity matrix of the occupation indices of the states across individuals was sorted using modularity optimization. The children with ADHD-C were labeled with “A,” and the TDC were labeled with “T.” The last three lows (red labels) indicate CBCL-EXT, CBCL-INT, and CBCL-AP. **(B)** The occupation indices of the three major CSD for each subtype C1, C2, and C3 are displayed. **(C)** The occupation indices of the three dominant states of individuals (S2, S4, and S5) were plotted with colors (subtype C1 with red, subtype C2 with blue, and subtype C3 with green).

Using the HMM-MAR results, we calculated the occupation indices (the portion of samples belonging to each state for the whole time series) for the five functional connectivity states (S1–S5) during the whole time series (*n* = 360). According to the occupation index for the five states, we created a distance matrix across the individuals. The distance between two individuals was measured by the sum of the absolute differences between the occupation indices of the two individuals for the five states at all 21 windows. We conducted modularity optimization (Newman, 2006) and found three modules (subtypes) across individuals (Figure 6). Children in the minor module (*N* = 3) were excluded in the subsequent analysis. Children with ADHD-C were found in all three clusters with similar ratios (61–70%); 22, 19, and 14 children with ADHD-C among 36, 29, and 20 total children in the clusters 1 (C1), 2 (C2), and 3 (C3), respectively.

When we compared the behavior scores of the ADHD-C in each subtype group, we found a significant difference between subtypes. The children with ADHD-C in C3 have higher CBCL-EXT scores (mean 67.8 and SD 7.7 for C3 and mean 58.8 and SD. 8.5 for C2) than the children with ADHD-C in C2 (*p* = 0.005) and has higher CBCL-AP scores (mean 75.2 and SD 11.5 for C3 and mean 68.5 and SD 6.5 for C1) than ADHD-C children in C1 (*p* = 0.038). No subtypes were disease-specific: the numbers of children in both ADHD-C and TDC were distributed almost equally for each subtype. Nevertheless, the subtyping of individuals by the major state occupation criteria was highly associated with the ADHD-C behavior scores (CBCL-EXT and CBCL-AP).

The correlation analyses result were between the elements of the state transition matrices and the ADHD behavior scores (CBCL-EXT, CBCL-INT, and CBCL-AP) and were between the ADHD behavioral scores and the occupation index of each state at each subtype were summarized in Table 1. Subtype-dependent correlations between state occupation metrics and ADHD symptom scores were observed; in C2 of ADHD-C, CBCL-EXT was positively correlated with occupation time of S5 and negatively with the occupation time of S4. This characteristic was not found in other subtypes.

**Table 1:** Correlation analysis results.

**Group**	**Variables**	**Correlation (*p*)**	**Group**	**Cluster**	**Variables**	**Correlation (*p*)**
ALL (*N* = 87)	A12 ~ EXT	0.220 (0.045)	ALL	Ca (*N* = 85)	EXT ~ OS5	0.258 (0.020)
	A12 ~ AP	0.306 (0.005)			AP ~ OS2	−0.304 (0.006)
	A21 ~ EXT	0.240 (0.028)			AP ~ OS5	0.249 (0.025)
	A21 ~ AP	0.334 (0.002)		C2 (*N* = 31)	AP ~ OS2	−0.455 (0.015)
	A22 ~ AP	−0.287 (0.008)			EXT ~ OS5	0.469 (0.012)
	A33 ~ AP	0.218 (0.046)	ADHD-C	Ca (*N* = 55)	EXT ~ OS4	−0.342 (0.013)
	A55 ~ EXT	0.257 (0.018)			EXT ~ OS5	0.290 (0.037)
	A55 ~ AP	0.244 (0.025)		C2 (*N* = 21)	EXT ~ OS4	−0.478 (0.045)
ADHD-C (*N* = 56)	A12 ~ AP	0.381 (0.005)			EXT ~ OS5	0.474 (0.047)
	A15 ~ EXT	0.271 (0.050)	TDC	C1 (*N* = 14)	AP ~ Entropy	0.636 (0.048)
	A15 ~ AP	0.359 (0.008)		C2 (*N* = 10)	EXT ~ OS5	0.798 (0.031)
	A21 ~ EXT	0.279 (0.043)				
	A21 ~ AP	0.401 (0.003)				
	A44 ~ EXT	−0.330 (0.016)				
	A52 ~ EXT	0.323 (0.018)				
	A55 ~ EXT	0.305 (0.026)				

*EXT, CBCL externalizing problems score; AP, CBCL attention problems score. A is the transition count matrix; e.g., A12 indicates the count of transitions from state 1 to state 2. The C1, C2, and Ca indicate clusters (subtypes) 1, 2, and all subtypes. OSn, occupation index of state n. Entropy, entropy of the occupation indices of all states in each individual. Children who do not have the corresponding behavior score were excluded from the correlation analysis (e.g., N = 87 for all children in the correlation analysis of EXT and AP). All p-values are uncorrected*.

**Figure 7:**
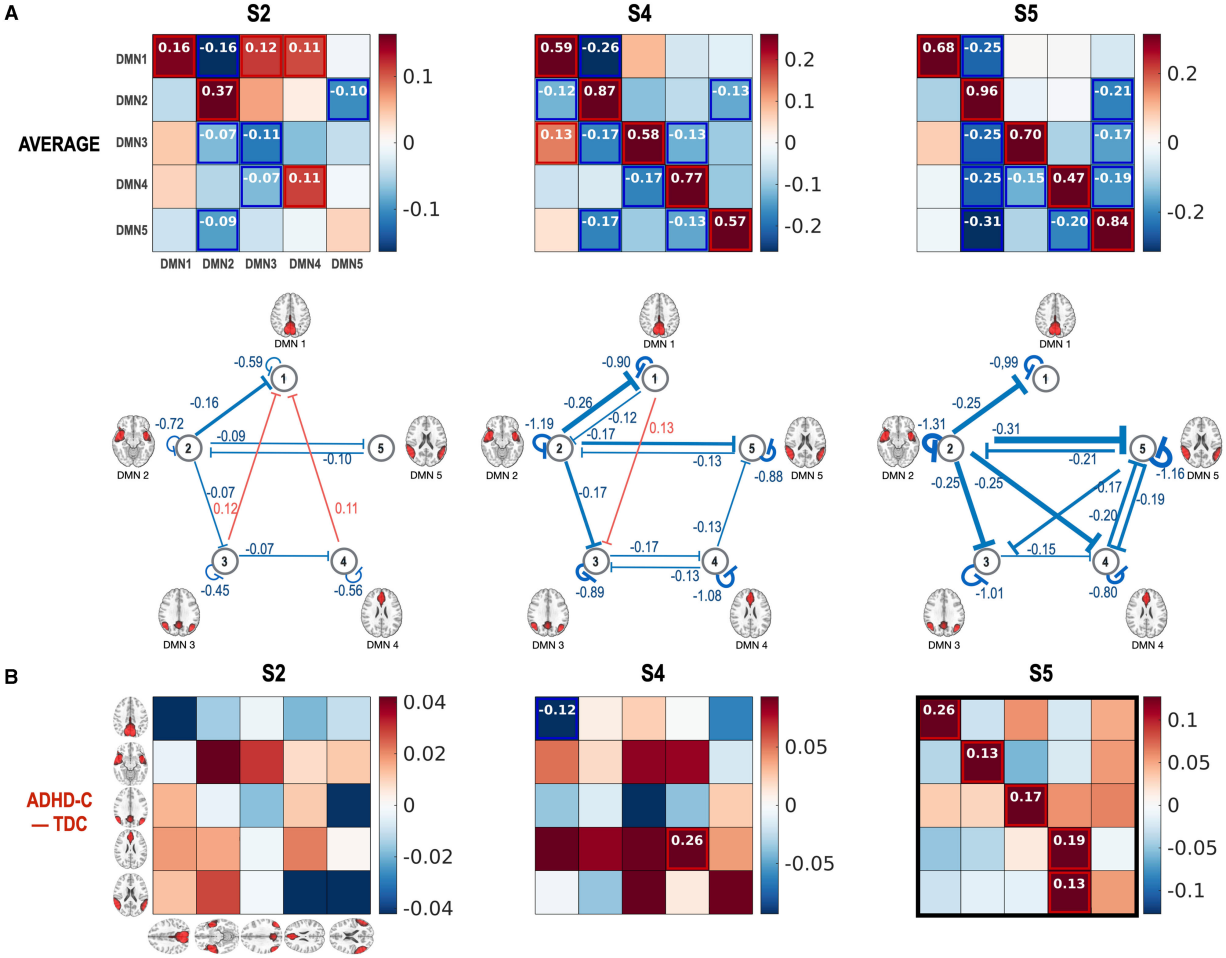
Group PEB results for the state-dependent effective connectivity. Group-level state-dependent effective connectivity matrices, and their connection maps were displayed for connectivity states S2, S4, and S5. A fixed-effects model (concatenating all DCMs for all children) was used for all the subjects (both ADHD-C and TDC) using PEB. The fixed-effect models for ADHD-C and TDC's spDCMs were hierarchically modeled with group common (average) **(A)** and group difference **(B)** design matrix using an additional PEB analysis step. We found none or few group differences in the effective connectivity at the states S2 and S4 while stronger self-inhibition was found in the ADHD-C compared with TDC in the state S5. The colors in the rectangular matrix represent the MAP for the connectivity from the column element to the row element. The diagonal term in the rectangular matrix should be interpreted after a transformation of −0.5 exp(A_ii_), to constrain the diagonal term for the self-connectivity to be inhibitory. For clarity, diagonal elements are displayed with transformed values in the network diagram in the second row of **(A)**. Effect sizes that survived a criterion of 95% posterior confidence are shown in numbers and rectangles.

Figure 7 presents the state-dependent effective connectivity in the group level of ADHD-C and TDC. For the group-level analysis, we conducted two levels of PEBs—the second-level PEB with the group common (average across groups) and group difference using X2 = [1 1; 1 −1] for the first-level PEB of the concatenated spDCMs of all children with ADHD-C and the first-level PEB of the concatenated spDCMs of all the TDC. Regardless of the functional connectivity states, DMN1 and DMN3 located at the precuneus and parietal lobules have positive couplings, while most causal couplings are inhibitory. Generally, ADHD-C has stronger inhibitory self-connection than TDC, in particular at the functional connectivity state 5 (Figure 7B).

**Figure 8:**
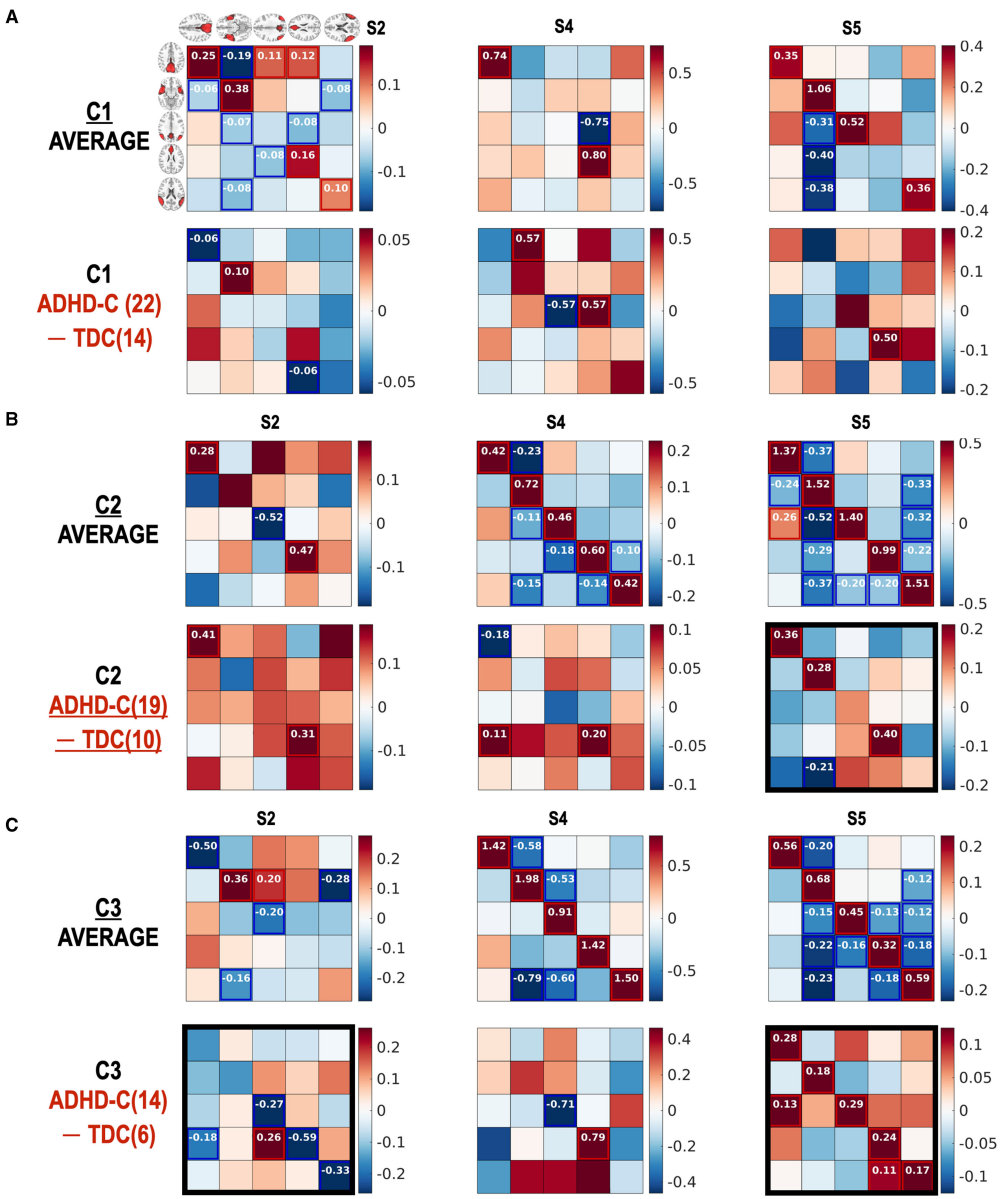
(Diagnostic) group comparison results for individuals in the same subtype according to the state occupation patterns. The group comparison results between ADHD-C and TDC according to subtype C1 **(A)**, subtype C2 **(B)**, and subtype C3 **(C)** are presented. Number in () indicates the number of subjects. We found significant (diagnostic) group differences in the effective connectivity at the functional connectivity states S2 and S5, particularly in the subtype group C3. Subtype-specific group differences between ADHD-C and TDC were detected. The diagonal term should be understood after a transformation of −0.5exp(A_ii_), indicating more self-inhibition for higher values of diagonal terms. The black rectangles show prominent group differences.

**Figure 9:**
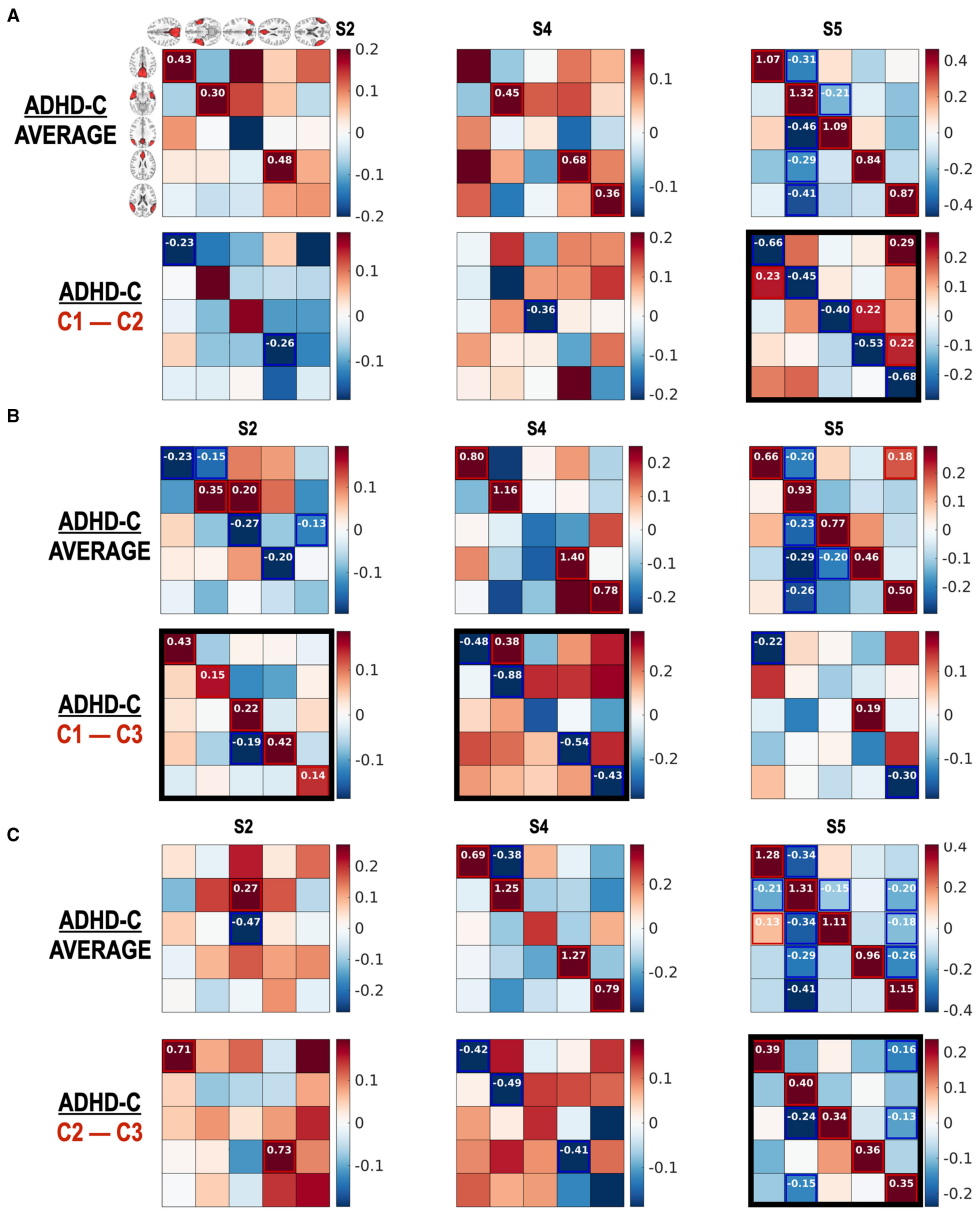
**(A–C)** Subtype-dependent effective connectivity differences in ADHD-C. According to functional connectivity states, children with ADHD-C have different effective connectivity, in particular between subtype groups C2 and C3 in state 5. See black rectangles that show obvious subtype differences.

**Figure 10:**
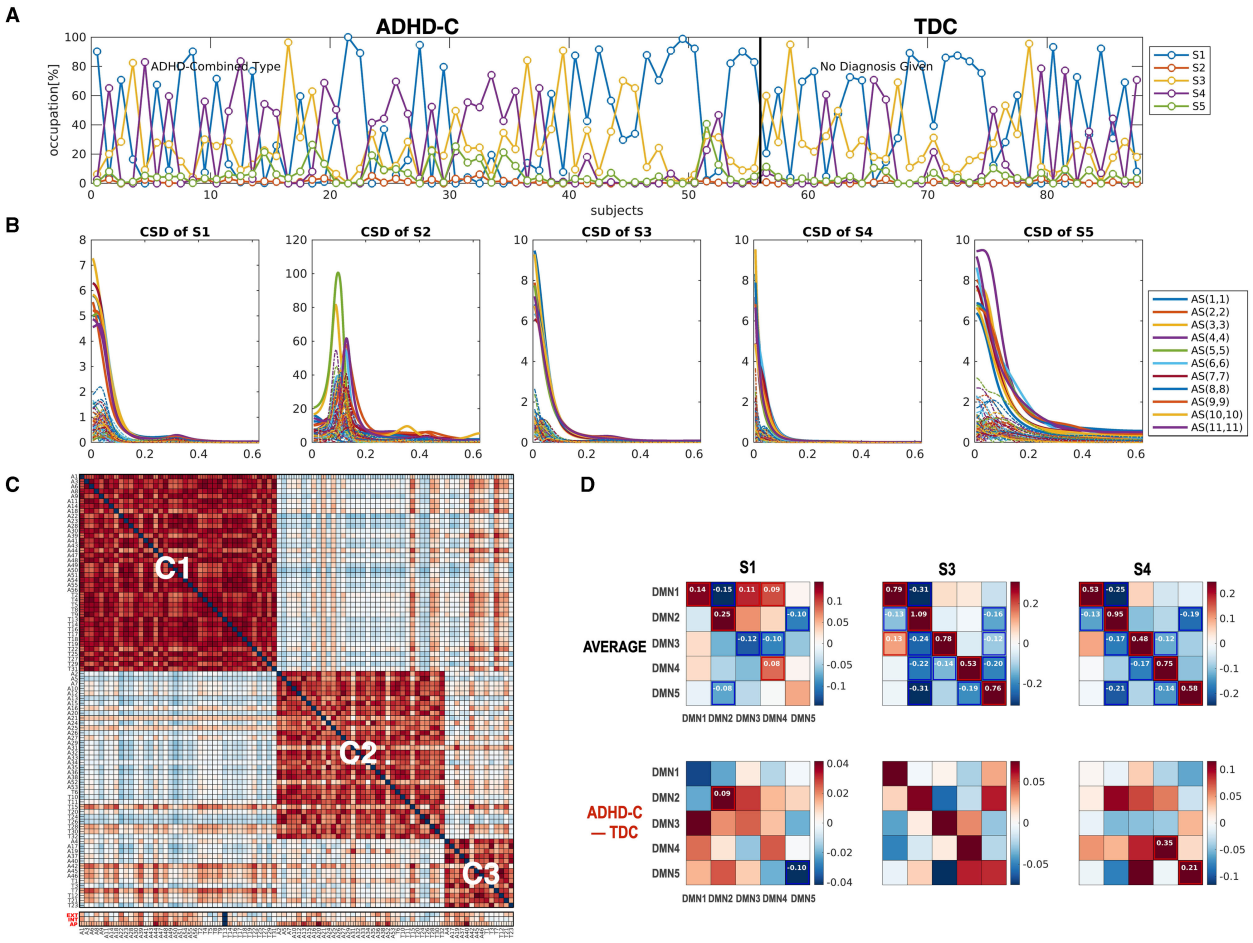
The state-dependent connectivity at the DMN with the whole brain connectivity as a state sequence. **(A)** The state distribution according to the whole-brain functional connectivity occupation indices and **(B)** the cross-spectra for each state, derived from the HMM-MAR of the whole brain time series, are displayed. **(C)** The modularity optimization of the similarity matrix of individuals according to the state occupation indices of the whole brain clustered individuals into three subtypes regardless of diagnostic groups. **(D)** The group average and group-difference in the state-dependent effective connectivity at the DMN (ADHD-C – TDC) are presented to show the state-dependent DMN effective connectivity according to the state of the whole-brain connectivity. Not many group differences exist for state-dependent connectivity when ADHD-C and TDC are analyzed as a whole. This calls for a subtype-level comparison of the groups, as shown in Figures 8, 9.

Figure 8 reports how the children with ADHD-C and TDC in the same subtype differ in the state-dependent effective connectivity. Meanwhile, Figure 9 presents how the children diagnosed with ADHD-C have different state-dependent effective connectivity according to the subtypes. We present Figure 10 to show the flexibility of the current state-dependent DCM in incorporating the diverse definition of brain states. For example, whole-brain connectivity states were used to explore the state-dependent effective connectivity in the DMN, which was explained in Figure 10.

## Discussion

The brain at rest is transitioning among multiple states. The transitions of the functional connectivity states have been associated with cognition or mental symptoms (for review, refer to Preti et al., 2017). For example, spontaneous switching between the states of functional connectivity is associated with cognitive performance in healthy older adults (Cabral et al., 2017). Vidaurre et al. (2017) reported cyclic transitions among two distinct sets of networks, and the relative occupation time was a subject-specific measure associated with cognitive traits. Alterations in time-varying functional connectivity states and their transitions are reported in mental diseases such as schizophrenia (Damaraju et al., 2014), bipolar disorders (Rashid et al., 2014), autism (De Lacy et al., 2017), and ADHD (De Lacy and Calhoun, 2018). Other studies have detected sleep and wakefulness using dynamic functional connectivity as a state (Damaraju et al., 2018). In the current study, we showed that the occupations of the dominant functional connectivity states could characterize individuals or subtypes of the same diagnostic group. According to major states defined with the cross-spectra, we were able to identify the effective connectivity responsible for group differences according to the brain states and subtypes in children with ADHD-C and TDC. Shappell et al. (2021) discovered that children with ADHD have a longer dwell time in the hyperconnected network states than healthy controls, using hidden semi-Markov models. In contrast, we did not find a significant difference in the state dwell time in the ADHD-C group compared with TDC when we defined the brain states according to the fifth-order MAR. However, we found that ADHD-C and TDC are composed of heterogeneous subtypes in the occupation times of major states, which show differential correlations between ADHD symptom scores and the occupation times of specific states according to the subtyped groups. The occupation patterns of the major connectivity states may be a new neural dimension independent of the axis of the symptom-based diagnosis of children.

From a technical perspective, the current study extends the dynamic effective connectivity analysis (Park et al., 2018). In Park et al. (2018), the dynamics of effective connectivity were modeled with the regression of multiple basis functions, either by data-independent, such as the discrete cosine transformation, or data-driven analyses, such as the principal component eigenvariates of the estimated effective connectivity series. The parameters for basis functions were estimated using PEB, which was further evaluated across groups. The concept of state-dependent effective connectivity is not new. Zarghami and Friston (2020) estimated both the state-dependent effective connectivity and transition parameters at a single loop of Bayesian parameter estimation by assigning an itinerant prior to the transition matrix. In their study, they focused on the temporal evolution of self-connection. In terms of model complexity, a current method is a simplified approach to model the brain dynamics using brain states determined outside the DCM framework. It is also flexible in incorporating the diverse definition of brain states in the model, for example, regional connectivity states or whole-brain connectivity states, as was exemplified in this study. The proposed method takes advantage of the hierarchical DCM framework, such as the Bayesian model reduction at the first level (effective connectivity estimation) and second level (parameters for state regressors), or the Bayesian model comparison (Friston et al., 2016). In the hierarchical DCM framework, a Bayesian model reduction from the full model (the fully connected model in the first level) can be achieved by changing the prior, i.e., shrinking model parameters and evaluating the posterior directly without re-estimation of effective connectivity using the spDCM. This Bayesian model reduction scheme can be effectively used in hierarchical model inversion, making the second- or third-level PEB analysis possible, without recalculating the first-level model inversion using spDCM (Friston et al., 2015, 2016). The hierarchical model reduction could also be used in the second-level PEB analysis in finding optimal state regressors.

The current study highlights the importance of subtyping individuals according to connectivity state—subtyping which reveals effective connectivity differences between diagnostic groups, which would otherwise go undetected. As shown in Figure 7B, no significant group differences were found in state S2 without state occupation-based subtyping. However, when we explored the group differences in detail according to subtypes (Figure 8), decreased self-inhibition was found at the state S2 of ADHD-C in subtype C3 compared with the TDC of the same subtype. The increased self-inhibition at the state S5 of ADHD-C compared with the TDC may be associated with the same phenomenon found in the subtype C3 (Figure 8C). Most of the DMNs of ADHD-C have a higher inhibitory self-connection than TDC at state S5. The importance of self-inhibitory connection has been dealt with in various studies, the winnerless competition in the brain (Zarghami and Friston, 2020) and seizure onset (Papadopoulou et al., 2015). An increase in self-inhibition corresponds to a reduction in the excitability of the neuronal populations to their afferents (and recurrent self connections). This may be particularly important in the present setting because many formulations of psychopathology focus on the sensitivity or excitability of neuronal populations (Pellicano and Burr, 2012; Fitzgerald et al., 2015; Ainley et al., 2016; Friston, 2017; Powers et al., 2017; Parr et al., 2018). For example, in the research on schizophrenia and autism, much attention is paid to excitation-inhibition balance (Lisman, 2012; Wang and Krystal, 2014). Computational accounts under predictive coding interpret these changes in excitability as a failure to attenuate or modulate the precision of prediction errors; namely, the postsynaptic sensitivity of neuronal populations thought to encode prediction errors (e.g., superficial pyramidal cells) (Bastos et al., 2012; Shipp, 2016). In these accounts, the ensuing psychopathology is often related to an imbalance between the sensory and prior precision at the lower and higher levels in the cortical hierarchy, respectively. The current study further details the critical role of the self-inhibitory connectivity at a specific brain state according to subtypes of ADHD-C.

The current study also argues the heterogeneity of effective connectivity in ADHD-C. All the children with ADHD-C do not have the same neural circuitry, which was revealed by comparing the effective connectivity differences between the subtype groups for the same diagnosis (Figure 9). The ADHD-C shows significant subtype differences between the subtype groups C1 and C3 for the dominant state S2. At the state S5, the subtype group C2 showed an increased self-inhibition compared with the subtype groups C1 and C3 of ADHD-C. State S4 of ADHD-C shows a subtype-dependent effective connectivity difference between the C1 and C3 groups, where increased self-inhibition was found in the C3 group compared with the C1 group. The subtype differences are not surprising as we divided the subtypes of a diagnostic group according to the occurrences of the dominant cross-spectra or functional connectivity pattern of either ADHD-C or TDC. However, by conducting an effective connectivity estimation, we capture the details of the brain interactions among the brain regions of the DMN that generate diverse functional connectivity. Although the subtyping was conducted according to the occupation of neural connectivity states, a significant difference of ADHD-related behavior scores exists between the subtypes of ADHD-C; there are more serious externalizing problems in group C3 than in C2 and higher attention problems scores in group C3 than in C1. The state occupation indices are also associated with the ADHD-C behavior scores (CBCL-EXT and CBCL-AP) (See Table 1). These results imply the potential roles of the occupation of the dominant DMN functional connectivity states as biomarkers of ADHD-related symptoms.

All these empirical results suggest the need for state-dependent and subtype-dependent effective connectivity analyses due to the heterogeneous distribution of ADHD-C and TDC regarding dominant functional connectivity patterns (fast dynamics) and their occurrence patterns (slow dynamics).

The brain has multiple time scales of neural dynamics (Zhigalov et al., 2015; Yano et al., 2016; Williams et al., 2018; Koksal Ersoz et al., 2020; Spitmaan et al., 2020). In the multiple time scale perspective, we can view the current framework as a method to explore the slow dynamics of the fast time scale neural dynamics. The slow time scale dynamics refer to the state transitions in HMM, while fast time scale dynamics reflect diverse types of synchrony across the brain regions, i.e., functional connectivity detected in the MAR. We subtyped individuals according to the characteristic of both the slow and fast dynamics, i.e., the state occupation pattern and the functional connectivity (cross-spectra) pattern. Note that we used MAR in the HMM framework (equivalent to CSD) to define functional connectivity states. In the spDCM, effective connectivity was derived by inverting a forward function between the effective connectivity and CSD (Friston et al., 2014)—spDCM optimizes the model parameters (effective connectivity) to fit model-generated CSD and empirical CSD at each window. Thus, the MAR of the rsfMRI contains information corresponding to effective connectivity of the neural circuitry, richer than simple Pearson-correlation coefficients.

In defining a brain state, one may cluster the effective connectivity of each window and utilize the cluster indices for all windows as state regressors in the PEB to identify the state-dependent effective connectivity. This approach can be a categorical version of the dynamic effective connectivity study (Park et al., 2018), where the eigenvariates (after PCA) of the estimated effective connectivity sequence were again used to infer the dynamic components of effective connectivity. Besides the easy definitions of diverse brain states, the current approach using HMM has an advantage for the regressor-based PEB analysis. Most dynamic connectivity analyses based on sliding windows have assigned a state to each window as a winner-take-all scheme even when a window is composed of several connectivity states. This cannot differentiate some windows with slightly different portions of states. To resolve this, we incorporated the amount of each state for each window to the PEB regressor, which was possible by estimating the brain state at each time point using HMM. This is one of the critical concepts of the current study using HMM because the spDCM is estimated on regularly spaced windows, and multiple brain states within a window should be differently weighted in the PEB regression. This continuous weighting may make the state-dependent effective connectivity analysis using HMM-MAR comparable with or advantageous over the clustering-based state-dependent effective connectivity analysis in a simulation setting (Supplementary Appendix 2). However, we cannot generalize it based on a simple simulation result, and more intensive studies with diverse experimental data are necessary.

In addition to the state definition issue of multiple states within a window, other technical issues exist regarding the window size or overlap selection for the spDCM estimation. A small window size may capture fast state transitions; however, it may not provide a robust fit for the spDCM, as Zarghami and Friston (2020) discussed. Thus, the window size and overlap size of the dynamic effective connectivity analysis should be chosen to balance the parameter estimation accuracy and sensitivity to neural fluctuations in empirical data. However, it is not trivial to evaluate both estimation accuracy and the properties of neural fluctuations, even in the current Bayesian framework. If the number of windows differs due to different window sizes, the data size and regression length for the second level model also vary for different window sizes. Since the model and data themselves differ with the number of windows, the direct comparison of model evidence is inappropriate in the PEB framework. Zarghami and Friston (2020) proposed a novel criterion to determine the optimal window size by maximizing the relative log evidence of a dynamic state model compared with a stationary model for a given window size. The stationary model assigns only a single state for the whole time series. This approach chooses a window size that maximizes the log BF (Kass and Raftery, 1995). In the current experimental data, the window size 60 and overlap size 45 was an optimal choice in terms of the model evidence gain over the stationary model. The window size of 60 samples (48 s) and an overlap of 45 samples (36 s) falls within the range which is conventionally used in dynamic functional connectivity studies (Savva et al., 2019; Zhuang et al., 2020). This window size was also reliable in the parameter estimation, as presented in Supplementary Appendix 1. Supplementary Appendix 1 explains the window-size dependent accuracy of spDCM for four nodes networks used in the simulation.

It should be noted that the window size (and overlap size) was chosen according to the model evidence gain over the stationary model of the given window size and overlap size. In contrast to the original study, the current simplified framework does not include the model for state transitions (Zarghami and Friston, 2020). Since the stationary model with different window sizes may not be the same in parameter estimation and in reflecting the same underlying neural fluctuations between windows and within windows (containing lower frequency band for longer window), the utility of the model evidence gain (i.e., log BF) in the optimal window size selection needs to be further elaborated. The current window size and overlap size, chosen according to the relative BF criterion in the present experimental data, might not be generalized for other studies. Alternative to finding an optimal window size that differs according to the criterion, we may select a specific window of interest according to the research hypothesis for a particular frequency band of underlying neural state transitions, particularly when the brain state transitions in multiple frequency bands. Analysis with longer windows would be sensitive to slow fluctuations, and shorter windows would provide more information regarding fast state transitions. Regardless of the window size selection method, either by optimization or hypothesis, the result should be interpreted in that frequency band: for example, a window size of 60 (48 s) and a 15 step size (12 s) presented in the current study.

In theory, the state-dependent effective connectivity analysis may be performed without windowing if we can partition the time series with change point detection algorithms (e.g., Jeong et al., 2016). Since we could use different regressors with ease, we mainly focused on the window-based regression analysis for simplicity purposes. We speculated that the weighed regressor using portions of the HMM states at the windows would be beneficial in making the state-dependent effective connectivity analysis less sensitive to the window size selection. The accuracy of HMM-MAR is another challenge for the reliable estimation of the state-dependent effective connectivity. We also have issues with the optimal choice of the state numbers and MAR order, which is a common difficulty encountered in the application of HMM-MAR (Vidaurre et al., 2017, 2018; Stevner et al., 2019). One of the most critical issues in clustering-based dynamic connectivity studies is the selection of maximal clusters *K*. In contrast with the conventional clustering approach, there is an additional constraint in choosing maximal clusters in the PEB model since a PEB with many regressors (each cluster membership is a regressor column) becomes unstable (too many parameters to estimate). Therefore, we begin with *K* = 5, which was conventionally used in dynamic functional connectivity studies (De Lacy and Calhoun, 2018). Among the five states, only three states were prevalent across individuals regardless of the group, while the two states minimally occur to the individuals in the current dataset. Modularity optimization according to the occupation patterns of the five states showed that three dominant individual subtypes are clustered according to the usage of the three primary states (Figure 6). Thus, we evaluated the remainder analysis with the three major states and used the occupation indices of those states as regressors.

Since spDCM accounts for hemodynamic delays in the model fitting but not HMM, one may raise a question on the timing differences between the spDCM parameters and HMM-based states as regressors. Since spDCMs were evaluated on regularly spaced windows and the same time-delayed data were used to estimate both spDCM parameters and HMM states for each window, the timing difference between spDCM parameters and HMM states—due to the inclusion of hemodynamic responses as parameters in the model (spDCM) or not (HMM)—may not be critical in the current framework. Nevertheless, one may consider the effect of hemodynamic response delay in selecting window size. The long window may be less affected by the previous state changes (if they exist) before the window.

In the current study, we used a fixed-effects model for the group-level analysis of ADHD-C and TDC. When all the subjects have all the major states, random-effects modeling could be applied. However, if some people do not have certain states, the group PEB of individualized PEBs is not trivial at the current stage. Although we mainly analyzed the DMN effective connectivity according to the interaction states at the DMN, we could also analyze the DMN effective connectivity with the states defined by the global interaction. The whole-brain interaction may play a neural context for the subsystem of the DMN. Further work could help elucidate the underlying interaction of the local and global networks.

Regarding the neurobiological interpretation of the ADHD-C vs. TDC results, several limitations may have affected our interpretation. We chose a subset of a large database to ensure homogeneity of the data in terms of the mental symptoms. We limited the most common type of ADHD (ADHD-C) at the early age of ADHD. We also limited the age range between 7 and 10 years to reduce age-related variations. This restriction makes the sample size relatively small. Since the TDC in the current study did not meet the gender imbalance found in the ADHD group, the current study should be interpreted in consideration of the gender bias. Furthermore, we did not have complete documentation of the history of stimulant use and the current medication status of the individual participants from the HBN database. A more detailed evaluation with a larger sample size would aid in interpreting the state-dependent effective connectivity in ADHD-C.

In conclusion, we demonstrated the utility of state-dependent analysis of the directed coupling in mental diseases by evaluating the state-dependent subtypes of ADHD-C and TDC and comparing the effective connectivity.

## Data Availability Statement

Publicly available datasets were analyzed in this study. The dataset was obtained from the Child Mind Institute's HBN Biobank (http://fcon_1000.projects.nitrc.org/).

## Ethics Statement

Ethical review and approval was not required for the study on human participants in accordance with the local legislation and institutional requirements. Written informed consent from the participants' legal guardian/next of kin was not required to participate in this study in accordance with the national legislation and the institutional requirements.

## Author Contributions

HJP developed the idea and method, and received funds. JK and JE performed computational simulations and analyses. HJP and JE performed the analysis with fMRI data. HJP and JK wrote the manuscript. JE, CP, SP, and JS prepared the data and contributed to test the scheme. All authors contributed to the article and approved the submitted version.

## Funding

This research was supported by the Brain Research Program and the Brain Pool Program through the National Research Foundation of Korea (NRF) funded by the Ministry of Science and ICT (NRF-2017M3C7A1049051 and NRF-2017M3C7A1030750) (HJP).

## Conflict of Interest

The authors declare that the research was conducted in the absence of any commercial or financial relationships that could be construed as a potential conflict of interest.

## Publisher's Note

All claims expressed in this article are solely those of the authors and do not necessarily represent those of their affiliated organizations, or those of the publisher, the editors and the reviewers. Any product that may be evaluated in this article, or claim that may be made by its manufacturer, is not guaranteed or endorsed by the publisher.

## References

[B1] Achenbach T. M. Rescorla L. A. (2001). Manual for the ASEBA School-Age Forms and Profiles: an Integrated System of Multi-Informant Assessment. Burlington: ASEBA.

[B2] Ainley V. Apps M. A. Fotopoulou A. Tsakiris M. (2016). ‘Bodily precision’: a predictive coding account of individual differences in interoceptive accuracy. Philos. Trans. R. Soc. Lond,. B,. Biol. Sci. 371:20160003. 10.1098/rstb.2016.0003 28080962 PMC5062093

[B3] Alexander L. M. Escalera J. Ai L. Andreotti C. Febre K. Mangone A.. (2017). An open resource for transdiagnostic research in pediatric mental health and learning disorders. Sci. Data 4:170181. 10.1038/sdata.2017.181 29257126 PMC5735921

[B4] Allen E. A. Damaraju E. Plis S. M. Erhardt E. B. Eichele T. Calhoun V. D. (2014). Tracking whole-brain connectivity dynamics in the resting state. Cereb. Cortex 24, 663–676. 10.1093/cercor/bhs352 23146964 PMC3920766

[B5] Anderson A. Douglas P. K. Kerr W. T. Haynes V. S. Yuille A. L. Xie J.. (2014). Non-negative matrix factorization of multimodal MRI, fMRI and phenotypic data reveals differential changes in default mode subnetworks in ADHD. Neuroimage 102(Pt 1), 207–219. 10.1016/j.neuroimage.2013.12.015 24361664 PMC4063903

[B6] Barber A. D. Jacobson L. A. Wexler J. L. Nebel M. B. Caffo B. S. Pekar J. J.. (2015). Connectivity supporting attention in children with attention deficit hyperactivity disorder. Neuroimage Clin. 7, 68–81. 10.1016/j.nicl.2014.11.011 25610768 PMC4299959

[B7] Bastos A. M. Usrey W. M. Adams R. A. Mangun G. R. Fries P. Friston K. J. (2012). Canonical microcircuits for predictive coding. Neuron 76, 695–711. 10.1016/j.neuron.2012.10.038 23177956 PMC3777738

[B8] Breakspear M. (2017). Dynamic models of large-scale brain activity. Nat. Neurosci. 20, 340–352. 10.1038/nn.4497 28230845

[B9] Brown M. R. Sidhu G. S. Greiner R. Asgarian N. Bastani M. Silverstone P. H.. (2012). ADHD-200 Global Competition: diagnosing ADHD using personal characteristic data can outperform resting state fMRI measurements. Front. Syst. Neurosci. 6:69. 10.3389/fnsys.2012.00069 23060754 PMC3460316

[B10] Cabral J. Kringelbach M. L. Deco G. (2014). Exploring the network dynamics underlying brain activity during rest. Prog. Neurobiol. 114, 102–131. 10.1016/j.pneurobio.2013.12.005 24389385

[B11] Cabral J. Vidaurre D. Marques P. Magalhaes R. Silva Moreira P. Miguel Soares J.. (2017). Cognitive performance in healthy older adults relates to spontaneous switching between states of functional connectivity during rest. Sci. Rep. 7, 5135. 10.1038/s41598-017-05425-7 28698644 PMC5506029

[B12] Calhoun V. D. Adali T. Pearlson G. D. Pekar J. J. (2001). A method for making group inferences from functional MRI data using independent component analysis. Hum. Brain Mapp. 14, 140–151. 10.1002/hbm.1048 11559959 PMC6871952

[B13] Calhoun V. D. Miller R. Pearlson G. Adali T. (2014). The chronnectome: time-varying connectivity networks as the next frontier in fMRI data discovery. Neuron 84, 262–274. 10.1016/j.neuron.2014.10.015 25374354 PMC4372723

[B14] Chang C. Glover G. H. (2010). Time-frequency dynamics of resting-state brain connectivity measured with fMRI. Neuroimage 50, 81–98. 10.1016/j.neuroimage.2009.12.011 20006716 PMC2827259

[B15] Cribben I. Haraldsdottir R. Atlas L. Y. Wager T. D. Lindquist M. A. (2012). Dynamic connectivity regression: determining state-related changes in brain connectivity. Neuroimage 61, 907–920. 10.1016/j.neuroimage.2012.03.070 22484408 PMC4074207

[B16] Damaraju E. Tagliazucchi E. Laufs H. Calhoun V. D. (2018). Connectivity dynamics from wakefulness to sleep. bioRxiv 380741. 10.1101/380741 PMC7753906 32562782

[B17] Damaraju E. Allen E. A. Belger A. Ford J. M. Mcewen S. Mathalon D. H.. (2014). Dynamic functional connectivity analysis reveals transient states of dysconnectivity in schizophrenia. Neuroimage Clin. 5, 298–308. 10.1016/j.nicl.2014.07.003 25161896 PMC4141977

[B18] De Lacy N. Calhoun V. D. (2018). Dynamic connectivity and the effects of maturation in youth with attention deficit hyperactivity disorder. Network neuroscience (Cambridge, Mass.) 3, 195–216. 10.1162/netn_a_00063 30793080 PMC6372020

[B19] De Lacy N. Doherty D. King B. H. Rachakonda S. Calhoun V. D. (2017). Disruption to control network function correlates with altered dynamic connectivity in the wider autism spectrum. Neuroimage Clin. 15, 513–524. 10.1016/j.nicl.2017.05.024 28652966 PMC5473646

[B20] Deco G. Jirsa V. K. (2012). Ongoing cortical activity at rest: criticality, multistability, and ghost attractors. J. Neurosci. 32, 3366–3375. 10.1523/JNEUROSCI.2523-11.2012 22399758 PMC6621046

[B21] Deco G. Tononi G. Boly M. Kringelbach M. L. (2015). Rethinking segregation and integration: contributions of whole-brain modelling. Nat. Rev. Neurosci. 16, 430–439. 10.1038/nrn3963 26081790

[B22] Fair D. A. Posner J. Nagel B. J. Bathula D. Dias T. G. Mills K. L.. (2010). Atypical default network connectivity in youth with attention-deficit/hyperactivity disorder. Biol. Psychiatry 68, 1084–1091. 10.1016/j.biopsych.2010.07.003 20728873 PMC2997893

[B23] Fitzgerald T. H. B. Moran R. J. Friston K. J. Dolan R. J. (2015). Precision and neuronal dynamics in the human posterior parietal cortex during evidence accumulation. Neuroimage 107, 219–228. 10.1016/j.neuroimage.2014.12.015 25512038 PMC4306525

[B24] Freyer F. Roberts J. A. Becker R. Robinson P. A. Ritter P. Breakspear M. (2011). Biophysical mechanisms of multistability in resting-state cortical rhythms. J. Neurosci. 31, 6353–6361. 10.1523/JNEUROSCI.6693-10.2011 21525275 PMC6622680

[B25] Freyer F. Roberts J. A. Ritter P. Breakspear M. (2012). A canonical model of multistability and scale-invariance in biological systems. PLoS Comput. Biol. 8:e1002634. 10.1371/journal.pcbi.1002634 22912567 PMC3415415

[B26] Friston K. Zeidman P. Litvak V. (2015). Empirical Bayes for DCM: a group inversion scheme. Front. Syst. Neurosci. 9:164. 10.3389/fnsys.2015.00164 26640432 PMC4661273

[B27] Friston K. J. (2017). Precision psychiatry. Biol. Psychiatry Cogn. Neurosci. Neuroimag. 2, 640–643. 10.1016/j.bpsc.2017.08.007 29560899

[B28] Friston K. J. Holmes A. P. Worsley K. J. Poline J. B. Frith C. D. Frackowiak R. S. J. (1995). Statistical parametric maps in functional imaging: a general linear approach. Hum. Brain Mapp. 20, 189–210. 10.1002/hbm.460020402

[B29] Friston K. J. Kahan J. Biswal B. Razi A. (2014). A DCM for resting state fMRI. Neuroimage 94, 396–407. 10.1016/j.neuroimage.2013.12.009 24345387 PMC4073651

[B30] Friston K. J. Litvak V. Oswal A. Razi A. Stephan K. E. Van Wijk B. C.. (2016). Bayesian model reduction and empirical Bayes for group (DCM) studies. Neuroimage 128, 413–431. 10.1016/j.neuroimage.2015.11.015 26569570 PMC4767224

[B31] Handwerker D. A. Roopchansingh V. Gonzalez-Castillo J. Bandettini P. A. (2012). Periodic changes in fMRI connectivity. Neuroimage 63, 1712–1719. 10.1016/j.neuroimage.2012.06.078 22796990 PMC4180175

[B32] Himberg J. Hyvärinen A. Esposito F. (2004). Validating the independent components of neuroimaging time series *via* clustering and visualization. NeuroImage 22, 1214–1222. 10.1016/j.neuroimage.2004.03.027 15219593

[B33] Hutchison R. M. Womelsdorf T. Gati J. S. Everling S. Menon R. S. (2013). Resting-state networks show dynamic functional connectivity in awake humans and anesthetized macaques. Hum. Brain Mapp. 34, 2154–2177. 10.1002/hbm.22058 22438275 PMC6870538

[B34] Insel T. R. Cuthbert B. N. (2015). Medicine. Brain disorders? Precisely. Science 348, 499–500. 10.1126/science.aab2358 25931539

[B35] Jang C. Knight E. Q. Pae C. Park B. Yoon S. A. Park H. J. (2017). Individuality manifests in the dynamic reconfiguration of large-scale brain networks during movie viewing. Sci. Rep. 7:41414. 10.1038/srep41414 28112247 PMC5256084

[B36] Jeong S. O. Pae C. Park H. J. (2016). Connectivity-based change point detection for large-size functional networks. Neuroimage 143, 353–363. 10.1016/j.neuroimage.2016.09.019 27622394

[B37] Kang J. Pae C. Park H. J. (2019). Graph-theoretical analysis for energy landscape reveals the organization of state transitions in the resting-state human cerebral cortex. PLoS ONE 14:e0222161. 10.1371/journal.pone.0222161 31498822 PMC6733463

[B38] Kass R. E. Raftery A. E. (1995). Bayes factor and model uncertainty. J. Am. Stat. Assoc. 90, 773–795. 10.1080/01621459.1995.10476572

[B39] Kelso J. A. (2012). Multistability and metastability: understanding dynamic coordination in the brain. Philos. Trans. R. Soc. Lond. B. Biol. Sci. 367, 906–918. 10.1098/rstb.2011.0351 22371613 PMC3282307

[B40] Koksal Ersoz E. Modolo J. Bartolomei F. Wendling F. (2020). Neural mass modeling of slow-fast dynamics of seizure initiation and abortion. PLoS Comput. Biol. 16:e1008430. 10.1371/journal.pcbi.1008430 33166277 PMC7676664

[B41] Li Y.-O. Adal,i T. Calhoun V. D. (2007). Estimating the number of independent components for functional magnetic resonance imaging data. Hum. Brain Mapp. 28, 1251–1266. 10.1002/hbm.20359 17274023 PMC6871474

[B42] Lisman J. (2012). Excitation, inhibition, local oscillations, or large-scale loops: what causes the symptoms of schizophrenia? Curr. Opin. Neurobiol. 22, 537–544. 10.1016/j.conb.2011.10.018 22079494 PMC3302967

[B43] Monti R. P. Hellyer P. Sharp D. Leech R. Anagnostopoulos C. Montana G. (2014). Estimating time-varying brain connectivity networks from functional MRI time series. Neuroimage 103, 427–443. 10.1016/j.neuroimage.2014.07.033 25107854

[B44] Newman M. E. (2006). Modularity and community structure in networks. Proc. Natl. Acad. Sci. USA. 103, 8577–8582. 10.1073/pnas.0601602103 16723398 PMC1482622

[B45] Papadopoulou M. Leite M. Van Mierlo P. Vonck K. Lemieux L. Friston K.. (2015). Tracking slow modulations in synaptic gain using dynamic causal modelling: validation in epilepsy. Neuroimage 107, 117–126. 10.1016/j.neuroimage.2014.12.007 25498428 PMC4306529

[B46] Park H. J. Friston K. (2013). Structural and functional brain networks: from connections to cognition. Science 342:1238411. 10.1126/science.1238411 24179229

[B47] Park H. J. Friston K. J. Pae C. Park B. Razi A. (2018). Dynamic effective connectivity in resting state fMRI. Neuroimage 180, 594–608. 10.1016/j.neuroimage.2017.11.033 29158202 PMC6138953

[B48] Park H. J. Pae C. Friston K. Jang C. Razi A. Zeidman P.. (2017). Hierarchical dynamic causal modeling of resting-state fMRI reveals longitudinal changes in effective connectivity in the motor system after thalamotomy for essential tremor. Front. Neurol. 8:346. 10.3389/fneur.2017.00346 28775707 PMC5517411

[B49] Parr T. Benrimoh D. A. Vincent P. Friston K. J. (2018). Precision and false perceptual inference. Front. Integr. Neurosci. 12:39. 10.3389/fnint.2018.00039 30294264 PMC6158318

[B50] Pellicano E. Burr D. (2012). When the world becomes 'too real': a Bayesian explanation of autistic perception. Trends Cogn. Sci. 16, 504–510. 10.1016/j.tics.2012.08.009 22959875

[B51] Powers A. R. Mathys C. Corlett P. R. (2017). Pavlovian conditioning-induced hallucinations result from overweighting of perceptual priors. Science 357, 596–600. 10.1126/science.aan3458 28798131 PMC5802347

[B52] Preti M. G. Bolton T. A. Van De Ville D. (2017). The dynamic functional connectome: state-of-the-art and perspectives. Neuroimage 160, 41–54. 10.1016/j.neuroimage.2016.12.061 28034766

[B53] Qiu M. G. Ye Z. Li Q. Y. Liu G. J. Xie B. Wang J. (2011). Changes of brain structure and function in ADHD children. Brain Topogr. 24, 243–252. 10.1007/s10548-010-0168-4 21191807

[B54] Rabinovich M. I. Varona P. (2011). Robust transient dynamics and brain functions. Front. Comput. Neurosci. 5:24. 10.3389/fncom.2011.00024 21716642 PMC3116137

[B55] Rashid B. Damaraju E. Pearlson G. D. Calhoun V. D. (2014). Dynamic connectivity states estimated from resting fMRI Identify differences among Schizophrenia, bipolar disorder, and healthy control subjects. Front. Hum. Neurosci. 8:897. 10.3389/fnhum.2014.00897 25426048 PMC4224100

[B56] Razi A. Friston K. (2016). The connected brain: causality, models, and intrinsic dynamics. IEEE Signal Process. Mag. 33, 14–35. 10.1109/MSP.2015.2482121

[B57] Saad J. F. Griffiths K. R. Korgaonkar M. S. (2020). A systematic review of imaging studies in the combined and inattentive subtypes of attention deficit hyperactivity disorder. Front. Integr. Neurosci. 14:31. 10.3389/fnint.2020.00031 32670028 PMC7327109

[B58] Savva A. D. Mitsis G. D. Matsopoulos G. K. (2019). Assessment of dynamic functional connectivity in resting-state fMRI using the sliding window technique. Brain Behav. 9:e01255. 10.1002/brb3.1255 30884215 PMC6456784

[B59] Shappell H. M. Duffy K. A. Rosch K. S. Pekar J. J. Mostofsky S. H. Lindquist M. A.. (2021). Children with attention-deficit/hyperactivity disorder spend more time in hyperconnected network states and less time in segregated network states as revealed by dynamic connectivity analysis. Neuroimage 229:117753. 10.1016/j.neuroimage.2021.117753 33454408 PMC7979530

[B60] Shipp S. (2016). Neural elements for predictive coding. Front. Psychol. 7:1792. 10.3389/fpsyg.2016.01792 27917138 PMC5114244

[B61] Sonuga-Barke E. J. Castellanos F. X. (2007). Spontaneous attentional fluctuations in impaired states and pathological conditions: a neurobiological hypothesis. Neurosci. Biobehav. Rev. 31, 977–986. 10.1016/j.neubiorev.2007.02.005 17445893

[B62] Spitmaan M. Seo H. Lee D. Soltani A. (2020). Multiple timescales of neural dynamics and integration of task-relevant signals across cortex. Proc. Natl. Acad. Sci. USA. 117, 22522–22531. 10.1073/pnas.2005993117 32839338 PMC7486728

[B63] Stephan K. E. Weiskopf N. Drysdale P. M. Robinson P. A. Friston K. J. (2007). Comparing hemodynamic models with DCM. Neuroimage 38, 387–401. 10.1016/j.neuroimage.2007.07.040 17884583 PMC2636182

[B64] Stevner A. B. A. Vidaurre D. Cabral J. Rapuano K. Nielsen S. F. V. Tagliazucchi E.. (2019). Discovery of key whole-brain transitions and dynamics during human wakefulness and non-REM sleep. Nat. Commun. 10:1035. 10.1038/s41467-019-08934-3 30833560 PMC6399232

[B65] Tognoli E. Kelso J. A. (2014). The metastable brain. Neuron 81, 35–48. 10.1016/j.neuron.2013.12.022 24411730 PMC3997258

[B66] Van De Steen F. Almgren H. Razi A. Friston K. Marinazzo D. (2019). Dynamic causal modelling of fluctuating connectivity in resting-state EEG. Neuroimage 189, 476–484. 10.1016/j.neuroimage.2019.01.055 30690158 PMC6435216

[B67] Vidaurre D. Abeysuriya R. Becker R. Quinn A. J. Alfaro-Almagro F. Smith S. M.. (2018). Discovering dynamic brain networks from big data in rest and task. Neuroimage 180, 646–656. 10.1016/j.neuroimage.2017.06.077 28669905 PMC6138951

[B68] Vidaurre D. Quinn A. J. Baker A. P. Dupret D. Tejero-Cantero A. Woolrich M. W. (2016). Spectrally resolved fast transient brain states in electrophysiological data. Neuroimage 126, 81–95. 10.1016/j.neuroimage.2015.11.047 26631815 PMC4739513

[B69] Vidaurre D. Smith S. M. Woolrich M. W. (2017). Brain network dynamics are hierarchically organized in time. Proc. Natl. Acad. Sci. USA. 114, 12827–12832. 10.1073/pnas.1705120114 29087305 PMC5715736

[B70] Wang X. J. Krystal J. H. (2014). Computational psychiatry. Neuron 84, 638–654. 10.1016/j.neuron.2014.10.018 25442941 PMC4255477

[B71] Williams A. H. Kim T. H. Wang F. Vyas S. Ryu S. I. Shenoy K. V.. (2018). Unsupervised discovery of demixed, low-dimensional neural dynamics across multiple timescales through tensor component analysis. Neuron 98, 1099–1115 e1098. 10.1016/j.neuron.2018.05.015 29887338 PMC6907734

[B72] Yano S. Maeda T. Kondo T. (2016). Slow dynamics perspectives on the embodied-brain systems science. Neurosci. Res. 104, 52–55. 10.1016/j.neures.2015.11.002 26643384

[B73] Zang Y. F. He Y. Zhu C. Z. Cao Q. J. Sui M. Q. Liang M.. (2007). Altered baseline brain activity in children with ADHD revealed by resting-state functional MRI. Brain Dev. 29, 83–91. 10.1016/j.braindev.2006.07.002 16919409

[B74] Zarghami T. S. Friston K. J. (2020). Dynamic effective connectivity. Neuroimage 207:116453. 10.1016/j.neuroimage.2019.116453 31821868

[B75] Zhigalov A. Arnulfo G. Nobili L. Palva S. Palva J. M. (2015). Relationship of fast- and slow-timescale neuronal dynamics in human MEG and SEEG. J. Neurosci. 35, 5385–5396. 10.1523/JNEUROSCI.4880-14.2015 25834062 PMC6705402

[B76] Zhuang X. Yang Z. Mishra V. Sreenivasan K. Bernick C. Cordes D. (2020). Single-scale time-dependent window-sizes in sliding-window dynamic functional connectivity analysis: a validation study. Neuroimage 220:117111. 10.1016/j.neuroimage.2020.117111 32615255 PMC7594665

