# Supplementary material for: State-Dependent Effective Connectivity in Resting-State fMRI
Source: Front Neural Circuits. 2021 Oct 27;15:719364. doi: 10.3389/fncir.2021.719364 (PMC8579116; doi:10.3389/fncir.2021.719364)
Supplement: Supplementary file 1 [file Presentation_1.pdf]

Supplemental Appendix

State-dependent effective connectivity in resting-state fMRI

Hae-Jeong Park[1,2,3,4+*], Jinseok Eo[2,3+], Chongwon Pae[1,2,3], Junho Son[2,3], Sung Min Park[1], and Jiyoung Kang[1,2,3*]

[1]Department of Nuclear Medicine, Department of Psychiatry, Yonsei University College of Medicine, Seoul, Republic of Korea

[2]Graduate School of Medical Science, Brain Korea 21 Project, Yonsei University College of Medicine, Seoul, Republic of Korea

[3]Center for Systems and Translational Brain Science, Institute of Human Complexity and Systems Science, Yonsei University, Seoul, Republic of Korea

[4]Department of Cognitive Science, Yonsei University, Seoul, Republic of Korea

Keywords: dynamic causal modeling, ADHD, resting state fMRI, dynamic connectivity, effective connectivity

Appendix A1: Model parameter estimation accuracy according to window size

To evaluate the proper length of window size for the spDCM in terms of accuracy, we simulated fMRI time-series data for 1500 samples (1200 secs) using three different effective connectivity used in the simulation (see Method). Setting window sizes as 30, 45, 60, 75, and 90, we sampled 30 sets of simulated fMRI signals for each window size. We performed spDCM for each window, and compared estimated effective connectivity with the ground truth. As increasing the window size, the root mean square errors (RMSEs) and correlation coefficients between estimated and ground truth effective connectivity were decreased and increased, respectively.

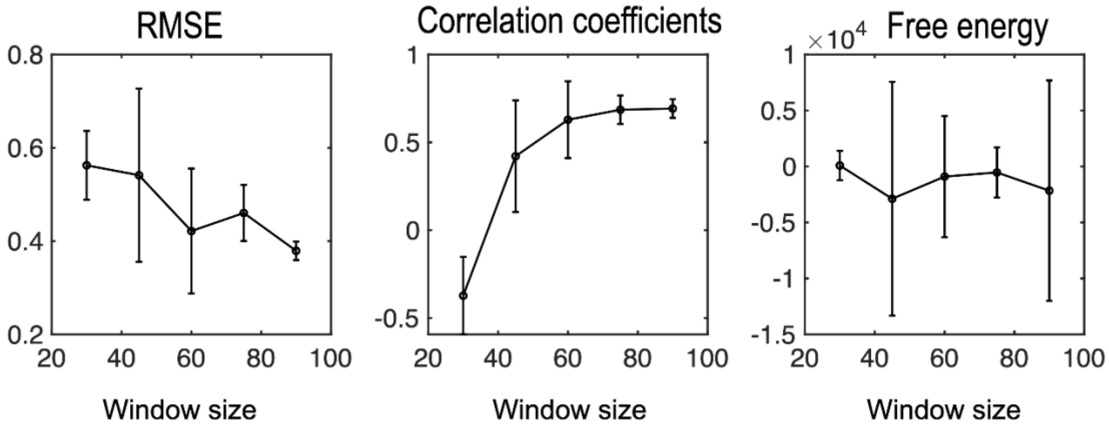

Figure A1. Window-size dependent accuracy in estimating effective connectivity using spDCM. The root mean square errors (RMSE), correlation coefficients between the estimated and the ground truth effective connectivity, and free energy are displayed. All points represent the average values of 30 windows (10 trials x 3 models) for each window size, and error bars indicate standard deviations.

Appendix A2: Simulation of state-dependent effective connectivity using K-means clustering of maximum a posterior probability (MAP) of effective connectivity

We performed a PEB analysis with a regressor obtained from k-means clustering of the estimated effective connectivity (MAP) at each window with the same simulation setting in the Method section. As a result, the correlation coefficients between the state-dependent effective connectivity and ground truth effective connectivity are r = 0.68, 0.61, and 0.74 (Figure A2B), which are similar to the results from HMM-MAR (r = 0.75, 0.6, and 0.75, Figure 3B). However, among 48 connectivity parameters, 26 effective connectivity of the ground truth (54%) were in the range of 95 % credible intervals of estimated effective

connectivity, which is fewer numbers than those in HMM-MAR results (38 effective connectivity, 80 %) presented in Figure 3.

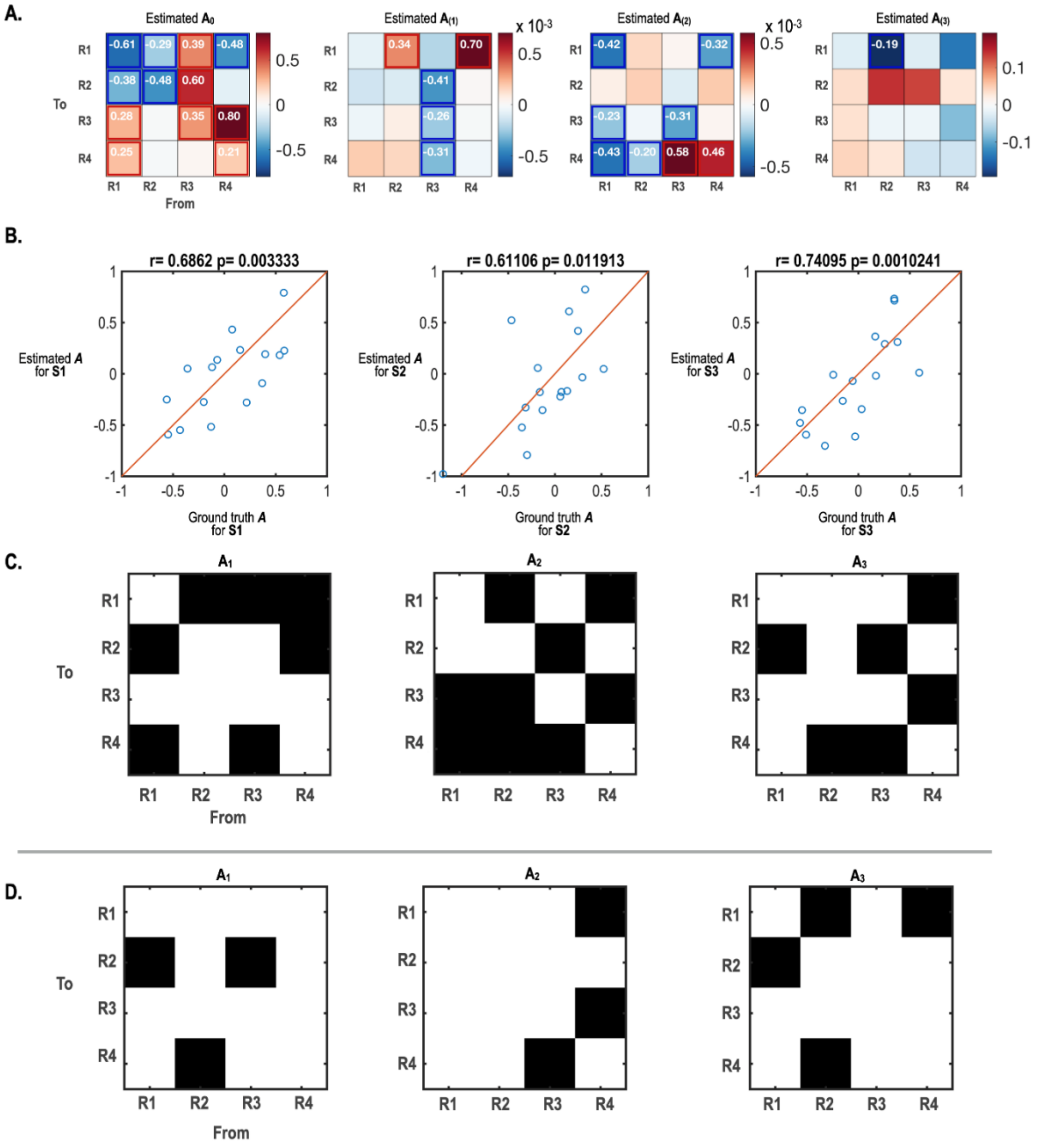

Figure A2. Simulation results of state-dependent effective connectivity estimation using K-means clustering of maximum a posterior probability (MAP) of effective connectivity. The same legend of Figure 3 can be applied to A) and B). C) and D) present black rectangles that the true connectivity is not in the range of 95 % credibility intervals. C) results from state-dependent effective connectivity analysis of using K-means clustering of MAP of effective connectivity, and D) results from state-dependent effective connectivity analysis of using HMM-MAR (the result presented in Figure 3). Among 48 network parameters, 26 parameters (the count of white rectangles) (~54 %) of true parameters were in the range of 95 % credible

intervals of estimated parameters in C); while 38 parameters (~80%) of true parameters were in the range of 95 % credible intervals of estimated parameters in D).
